# Alkyl 4-Aryl-6-amino-7- phenyl-3-(phenylimino)-4,7-dihydro- 3H-[1,2]dithiolo[3,4-b]pyridine-5-carboxylates: Synthesis and Agrochemical Studies

**DOI:** 10.3390/molecules28020609

**Published:** 2023-01-06

**Authors:** Victor V. Dotsenko, Anna E. Sinotsko, Vladimir D. Strelkov, Ekaterina A. Varzieva, Alena A. Russkikh, Arina G. Levchenko, Azamat Z. Temerdashev, Nicolai A. Aksenov, Inna V. Aksenova

**Affiliations:** 1 Department of Organic Chemistry and Technologies, Kuban State University, 350040 Krasnodar, Russia; 2 Department of Chemistry, North Caucasus Federal University, 355017 Stavropol, Russia; 3 Department of Analytical Chemistry, Kuban State University, 350040 Krasnodar, Russia

**Keywords:** active methylene thioamides, Michael addition, dithiomalondianilide, cyanoacetic esters, [1,2]dithiolo[3,4-b]pyridines, herbicide safeners

## Abstract

The reaction between dithiomalondianilide (N,N’-diphenyldithiomalondiamide) and alkyl 3-aryl-2-cyanoacrylates in the presence of morpholine in the air atmosphere leads to the formation of alkyl 6-amino-4-aryl-7-phenyl-3-(phenylimino)-4,7-dihydro-3H-[1,2]dithiolo[3,4-b]- pyridine-5-carboxylates in 37–72% yields. The same compounds were prepared in 23–65% yields by ternary condensation of aromatic aldehydes, ethyl(methyl) cyanoacetate and dithiomalondianilide. The reaction mechanism is discussed. The structure of ethyl 6-amino-4-(4-methoxyphenyl)-7-phenyl-3-(phenylimino)-4,7-dihydro-3H-[1,2]dithiolo[3,4-b]pyridine-5-carboxylate was confirmed by X-ray crystallography. Two of the prepared compounds showed a moderate growth-stimulating effect on sunflower seedlings. Three of the new compounds were recognized as strong herbicide safeners with respect to herbicide 2,4-D in the laboratory and field experiments on sunflower.

## 1. Introduction

**Scheme 1:**
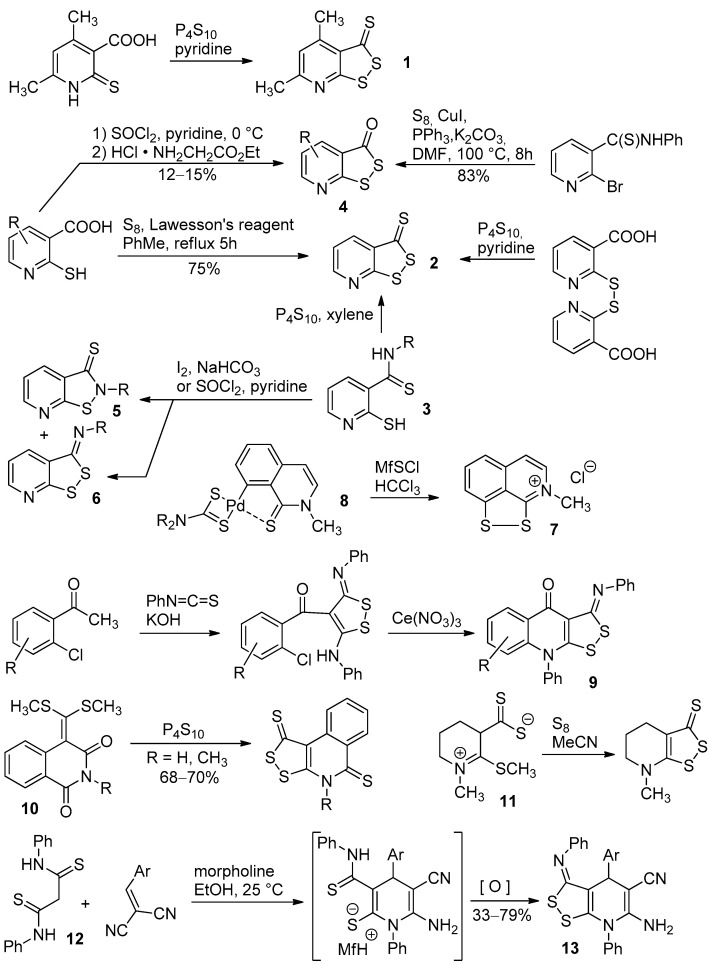
The reported methods [1,2,3,4,5,6,7,8,9,10,11,12,13,14,15,16] for construction of [1,2]dithiolo[3,4-b]pyridine system.

[1,2]Dithiolo[3,4-b]pyridine belongs to a rather rare class of heterocyclic systems. There are only a few papers that reported the preparation of such compounds. Historically, the first example of the synthesis is probably the preparation of dithiolopyridine **1** in low yield by reaction of 2-thioxonicotinic acid with phosphorus pentasulfide [1] (Scheme 1). Later the same approach was reported in other papers [2,3,4]. Compound **2** can also be prepared from 2-mercaptonicotinthioamides **3** upon treatment with P_4_S_10_ [4] or from 2,2’-dithionicotinic acid [5]. Dithiolopyridines **4** were synthesized in low yields by sequential treatment of 2-thioxonicotinic acid with thionyl chloride and then with ethyl glycinate [6] or from 2-bromo-N-phenylpyridine-3-thiocarboxamide and sulfur under Cu(I)-catalyzed cross-coupling conditions in 83% yield [7]. When nicotinthioamides **3** were treated with iodine or SOCl_2_/pyridine, mixtures of isothiazolopyridines **5** with isomeric 3-(R-imino)-[1,2]dithiolo[3,4-b]pyridines **6** were isolated [8]. The formation of dithiolopyridine **7** was observed when cyclopalladated complex **8** reacted with morpholinesulphenyl chloride (MfSCl) [9] (Scheme 1). [1,2]Dithioloquinolines **9** can be prepared by reaction of *ortho*-chloroacetophenones with phenyl isothiocyanate followed by Ce(NO_3_)_3_-catalyzed oxidative cyclization of the intermediate 4-(2-chlorobenzoyl)-5-(phenylamino)-3-(phenylimino)-3H-1,2-dithiols [10]. When treated with P_4_S_10_, ketene dithioacetals **10** afforded [1,2]dithioloisoquinolines in good yields [11]. 

The reactions of [1,2]dithiolo[3,4-b]pyridine **2** have also been studied in a limited number of papers [3,4,5,6,8,12,13,14]. Information about the synthesis and properties of partially saturated [1,2]dithiolo[3,4-b]pyridines is even more scarce. Thus, the only known synthesis of 4,5,6,7-tetrahydro[1,2]dithiolo[3,4-b]pyridine is not general and is based on the reaction of heterocyclic 1,4-dipol **11** with sulfur [15]. Finally, the reaction of dithiomalondianilide (N,N’-diphenyldithiomalondiamide) **12** with arylmethylene malononitriles yielded 4,7-dihydro[1,2]dithiolo[3,4-b]pyridines **13** [16] (Scheme 1).

Meanwhile, the antimicrobial, antitubercular [4] and antitumor [10] effects of [1,2]dithiolo[3,4-b]pyridines have been reported. Compounds of this type are of interest as starting reagents for the preparation of biologically active isothiazolopyridines and 2-mercaptonicotinthioamides [4,5,12,13]. As structural analogs and bioisosters of thieno[2,3-b]pyridines (for recent reviews see [17,18]), dithiolopyridines can be considered as promising molecules with still undiscovered potential.

In the context of our interest in exploring the chemistry of dithiomalondianilide **12** and its transformations leading to [1,2]dithiolo[3,4-b]pyridines [16,19,20,21], it seemed reasonable to study the reactions of dithiomalondianilide **12** with other Michael acceptors. It is noteworthy that despite the long-standing and active use of thioamide **12** as a bidentate S,S-chelating agent towards heavy metals (e.g., [22,23,24,25]), the heterocyclization reactions of **12** have been little studied to date [26,27,28,29]. 

This work presents the results of our studies of the reactions of N,N’-diphenyldithiomalondiamide **12** with 2-cyanoacrylates derived from cyanoacetic esters.

## 2. Results and Discussion

### 2.1. Synthesis

Earlier, we proposed a cascade method for the preparation of [1,2]dithiolo[3,4-b]pyridines **13** (Scheme 1) from N,N’-diphenyldithiomalondiamide **12** based on the morpholine-catalyzed Michael addition with arylmethylene malononitriles followed by heterocyclization and further air oxidation of 3-thiocarbamoylpyridine-2-thiolate intermediate [16]. As the continuation of this research, we have decided to look into the possibility to using other electron-deficient alkenes as Michael acceptors to prepare dithiolopyridines and we focused our attention on 3-aryl-2-cyanoacrylates **14**. The precursors **14** were prepared by Knoevenagel condensation of cyanoacetic esters with aromatic aldehydes in the presence of piperidine or morpholine. 

**Scheme 2:**
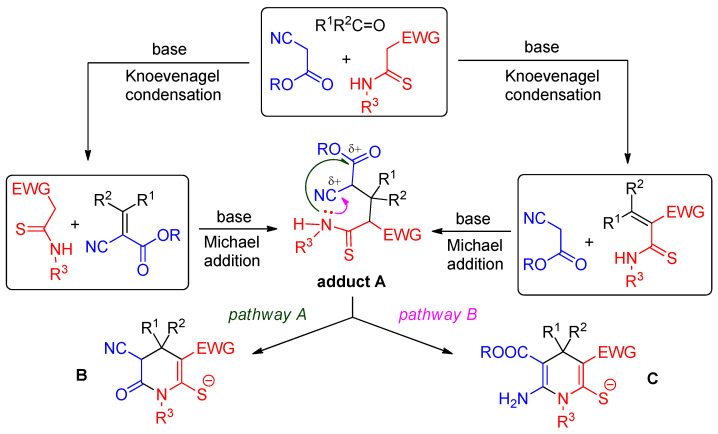
Two possible directions for heterocyclization reactions of active methylene thioamides with cyanoacetic esters or 2-cyanoacrylates.

Previous studies on the reactions involving cyanoacetates and active methylene thioamides and proceeding through the formation of Michael adducts **A** (Scheme 2) already showed that the further heterocyclization may follow two different reaction pathways. The first one suggests an intramolecular *6-exo-trig* process involving ester fragment COOR and leads to 3-cyanopyridin-2(1H)-ones **B** or related products (e.g., [30,31,32,33]). (pathway A, Scheme 2). An alternative path would be *6-exo-dig* heterocyclization, which leads to heterocyclic enamino esters **C** or its transformation products [34,35,36,37] (pathway B, Scheme 2).

**Scheme 3:**
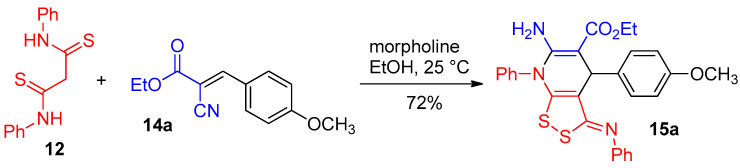
Synthesis of compound **15a**.

**Figure 1:**
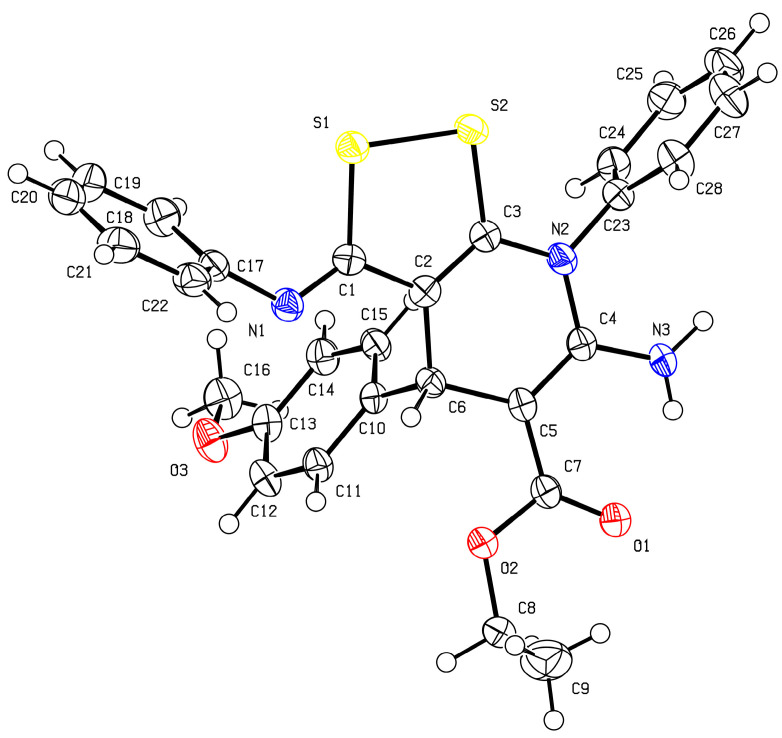
ORTEP drawing of the X-ray structure for ethyl 6-amino-4-(4-methoxyphenyl)-7-phenyl- 3-(phenylimino)-4,7-dihydro-3H-[1,2]dithiolo[3,4-b]pyridine-5-carboxylate **15a** (CCDC deposition number 2219352). Thermal ellipsoids for non-hydrogen atoms are shown at 50% probability. Blue: nitrogen, gray: carbon atoms, red: oxygen atoms, yellow: sulfur atoms.

First, when a mixture of N,N’-diphenyldithiomalondiamide **12** and (*E*)-ethyl 2-cyano-3-(4-methoxyphenyl)acrylate **14a** in EtOH were treated with excessive morpholine, a yellow crystal of ethyl 6-amino-4-(4-methoxyphenyl)-7-phenyl- 3-(phenylimino)-4,7-dihydro-3H-[1,2]dithiolo[3,4-b]pyridine-5-carboxylate **15a** was isolated in 72% yield (Scheme 3). The structure of **15a** was unambiguously confirmed by single crystal X-ray diffraction analysis (CCDC # 2219352, Figure 1).

**Scheme 4:**
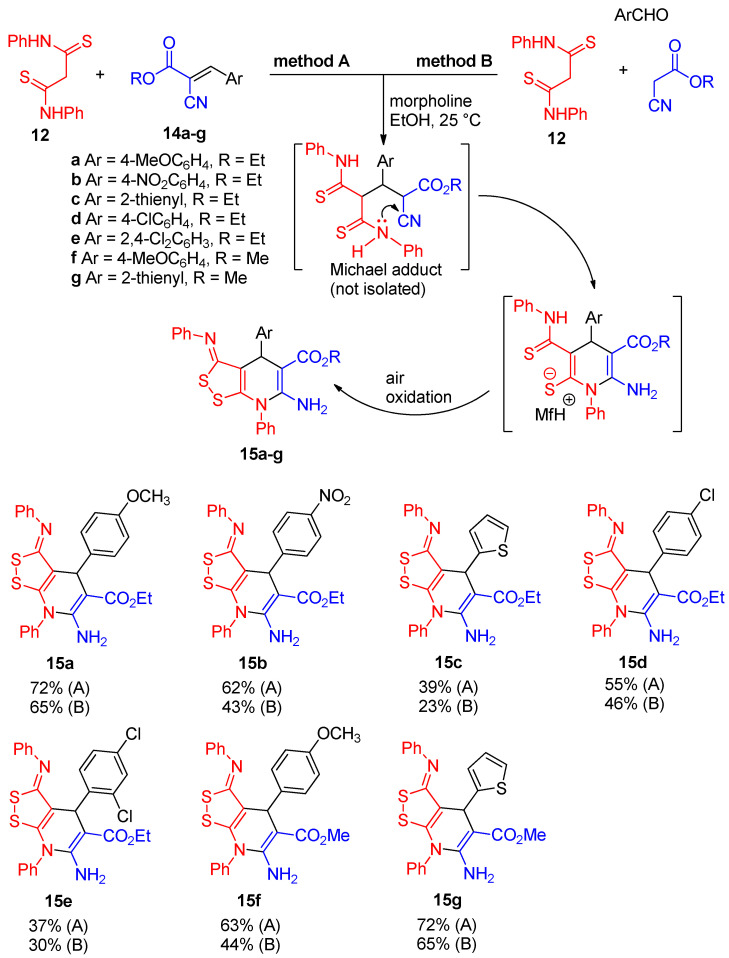
Synthesis of compounds **15a–g**.

At this point, some efforts to optimize the reaction conditions and examine the scope and limitation of the reaction were made. The reaction proceeded smoothly in MeOH or EtOH; other tested solvents (*i*-PrOH, acetone, DMF) gave rather unsatisfactory results. The nature of amine (morpholine, piperidine, triethylamine) does not significantly affect the yields of products. Along with Michael addition of **12** to other 2-cyanoacrylates **14a–g** (Method A, Scheme 4), we also investigated the three-component reaction of dithiomalondianilide **12**, cyanoacetic esters and aldehydes in the presence of morpholine (Method B, Scheme 4). However, Method B gives somewhat lower yields of dithiolopyridines **15**.

Extended refluxing of the reaction mixture does not favor the formation of dithiolopyridines **15**. Thus, when a mixture of **14a** and **12** in ethanol was heated under reflux in the presence of morpholine for as long as 15 h, a contaminated deep-brown material was obtained from which dithiolopyridine **15a** was isolated by recrystallization in only 13% yield. Less prolonged heating (1.5 h) was also accompanied by side processes and led to decreased yields; in this case, compound **15a** was obtained in 30% yield.

When the reaction of **12** with **14a** was conducted in inert atmosphere under nitrogen stream, dithiolopyridine **15a** was not isolated. This fact proves the crucial role of air oxygen for the final step of dithiolopyridine system formation. Nevertheless, the addition of oxidizing agents (hydrogen peroxide or iodine) leads to resinification of the reaction mixture. Aldehydes with both electron-donor and electron-withdrawing substituents are reacted well. However, we failed to obtain any products in the case of furfural or 2-cyano-3-(furan-2-yl)acrylates even if less nucleophilic and milder base triethylamine was taken instead of morpholine. This is probably due to the strong tendency of furan-ring-bearing electron-withdrawing substituents to undergo a nucleophilic attack at the C-5 position to form a complex mixture of furan-ring-cleavage/recyclization products.

In the IR spectra of compounds **15,** the absorption bands corresponding to NH_2_ valence vibrations (ν 3371–3474 cm^−1^ and ν 3263–3290 cm^−1^), RO(C=O) group bands (ν 1651–1661 cm^−1^) and imino group C=N–Ph bands at ν 1622–1638 cm^−1^ were observed while the bands corresponding to C≡N groups were absent. ^1^H NMR spectra of **15** revealed characteristic singlets attributed to H-4 protons at δ 5.02–5.94 ppm and a very broadened peak of NH_2_ protons at δ 7.00–7.19 ppm. It is interesting that in some cases the signal of OCH_2_ protons is detected not as a typical quartet but as a complex multiplet, probably due to the hindered rotation caused by the intramolecular hydrogen bond C=O...H-NH. Alternatively, the observed splitting of OCH_2_ signal can be caused by the shielding effect of the aromatic ring at C-4, which affects one of OCH_2_ protons. ^13^C NMR spectra lacked signals attributed to C=S carbons and revealed the peaks of C=O (δ 168.7–169.1 ppm), C=N (δ 163.0–163.4 ppm), C-3a (δ 111.1–113.7 ppm), C-5 (δ 76.5–78.4 ppm) and C-4 carbons (δ 33.5–38.6 ppm). The NMR, FTIR and HRMS spectra are shown in Supplementary Materials Figures S1–S6, S8–S31 and Tables S1, S8 and S9. The crystal data is shown in Supplementary Materials Tables S2–S7.

### 2.2. Agrochemical Studies

The new compounds were tested as herbicide safeners with respect to 2,4-dichlorophenoxyacetic acid (2,4-D) and as plant growth regulators. 2,4-D is an herbicide that is widely used for plant protection and was reported to show no significant toxicity to humans [38]. However, the use of 2,4-D has negative side effects, including its inhibition effect on the crops themselves that gives a decrease in yield by ~15–60%. To eliminate such negative effects and to raise crop yields, herbicide safeners (also called herbicide antidotes or detoxifiers) are successfully used. Herbicide safeners [39,40,41] can be defined as agrochemicals that are able to neutralize phytotoxins in plants, thus protecting crop plants from herbicide injury. Safeners are harmless to crop plants (or even have a growth-stimulating effect), but do not affect the activity of herbicides against weeds.

It is known that 3-aminothieno[2,3-b]pyridines, which can be considered as structural analogs of the prepared 3-imino-3H-[1,2]dithiolo[3,4-b]pyridines **15**, are reported to be effective herbicide safeners [42,43] and plant growth regulators [44]. We studied the efficiency of new 3-imino-3H-[1,2]dithiolo[3,4-b]pyridines as 2,4-D antidotes using sunflower seedlings using the reported procedure [42] (see also Materials and Methods). The antidote effect *A* was determined as a ratio of the hypocotyl (or root) length of sunflower seedlings in the “herbicide + antidote” experiments to the length in the reference group (where the seedlings were treated with 2,4-D only) (Equation (1)):

*A* = (*L*_exp_/*L*_ref_) × 100%, (1)

where *L*_exp_ is an organ length (mm) in the group of seedlings treated with herbicide and antidote, and *L*_ref_ is an organ length (mm) in the reference group of sunflower seedlings. 

We found that three of the new compounds, dithiolopyridines **15a,c,f**, exhibited a strong 2,4-D antidote effect in the laboratory experiments (Table 1). 

As we can see from the Table 1, the use of 3H-[1,2]dithiolo[3,4-b]pyridines **15a,c,f** as safeners against 2,4-D strongly reduce the toxic effects of the herbicide. Compounds **15a,c,f** reduced the negative effect of 2,4-D on sunflower seedling hypocotyls by 34–60% and by 40–55% on sunflower seedling roots.

The antidote activity of 3H-[1,2]dithiolo[3,4-b]pyridines **15a,c,f** was also studied in field experiments on sunflower in the experimental field of the Federal Scientific Center for Biological Protection of Plants (Krasnodar, Russia). Sunflower plants of cv. Master in the phase of 10–16 leaves were treated with an aqueous solution of 2,4-dichlorophenoxyacetic acid at a dose of 18 g/ha and 3 days later a safener solution was applied at a dose of 100 g/ha with the working fluid rate of 300 L/ha.

The field tests included the following variants:

- Control group—untreated plants;
- “Herbicide” (reference) group—plants treated with herbicide 2,4-D only;
- “Herbicide + antidote” group—plants treated with herbicide 2,4-D and an antidote.

Experiments were conducted in plots of 2.8 m^2^ with five-fold repetition. Sunflower harvesting was performed at the time of full seed maturity. The field antidote effect *A_F_* was determined by the absolute value of the crop yield increase to the herbicide reference by the Equation (2):

(2) $$A_{F}=\frac{Y_{1}-Y_{2}}{Y_{2}}\times 100\%,$$

where *A_F_* is antidote effect, %; *Y*_1_ is crop yield in “herbicide + antidote” group; and Y_2_ is crop yield in “herbicide” (reference) group.

The obtained data were statistically processed using Student’s *t*-test. The field test results are presented in Table 2. As it can be seen, the use of compounds **15a,c,f** as herbicide safeners under field conditions provides an antidote effect in the range of 41.4–51.4%.

The growth-stimulating activity of compounds **15a,c,f** was evaluated in laboratory experiments using the known procedure [45] on cv. Master sunflower seedlings (Table 3). The effect was evaluated by the elongation of stems and roots of treated seedlings in comparison to control (untreated seeds) (Equation (3)):

*E* = (*L*_treated_/*L*_control_) × 100%, (3)

where *E* is growth-stimulating effect, %; *L_treated_* is the length (mm) of stems/roots in the treated group of seedlings; and *L*_control_ is the length (mm) of stems/roots in the control (untreated) group of seedlings. 

As we can see from the Table 3, compounds **15a** and **15f** are more active than **15c** and favored stem elongation by 12–20% relative to control and stimulated root growth by 13–22%, depending on the concentration. In general, the growth-stimulating effect of compounds **15a** and **15f** can be considered as moderate.

The study of the acute toxicity of the new compounds is in progress. According to preliminary data, the compounds do not possess obvious phytotoxicity; this is indirectly indicated by the observed plant growth-stimulating effect of the tested samples (Table 3).

## 3. Materials and Methods

^1^H and ^13^C DEPTQ NMR spectra and 2D NMR experiments were recorded in solutions of DMSO-d_6_ on a Bruker AVANCE-III HD instrument (Bruker BioSpin AG, Fällanden, Switzerland) (at 400.40 or 100.61 MHz, respectively). Residual solvent signals were used as internal standards in DMSO-d_6_–2.49 ppm for ^1^H, and 39.50 ppm for ^13^C nuclei. Single crystal X-ray diffraction analysis of compound **15a** was performed on an automatic four-circle diffractometer Agilent Super Nova, Dual, Cu at zero, Atlas S2. HRMS spectra were recorded using a Bruker MaXis Impact quadrupole time-of-flight mass spectrometer (Bruker Daltonics, Bremen, Germany) equipped with an electrospray ionization source in positive ion detection mode. The voltage at the ionization source was 3.5 kV, the drying gas flow rate was 8 L/min, the spray gas pressure was 2 bar, the temperature of the ionization source was 250 °C, the mass scanning range (*m/z*) was 50–1000, the scanning speed was 3 Hz. The data were processed using Bruker Data Analysis 4.1 software. See Supplementary Materials File for NMR, FTIR and HRMS spectral charts and X-ray analysis data. 

FT-IR spectra were measured on a Bruker Vertex 70 instrument equipped with an ATR sampling module (Bruker Optics GmbH & Co. KG, Ettlingen, Germany). Elemental analyses were carried out using a Carlo Erba 1106 Elemental Analyzer (Carlo Erba Strumentazione, Cornaredo, Italy) Reaction progress and purity of isolated compounds were controlled by TLC on Sorbfil-A plates (Imid Ltd, Krasnodar, Russia), eluent–acetone:hexane 1:1 or ethyl acetate:light petroleum 3:1; the spots were visualized with UV-light and iodine vapors. N,N’-Diphenyldithiomalondiamide (dithiomalondianilide) **12** was prepared from acetylacetone and phenyl isothiocyanate as described earlier [19,46]. Aldehydes, cyanoacetic esters, morpholine and solvents were purchased from commercial vendors and purified by distillation (Galachem, Moscow, Russia).

**General procedure for the preparation of dithiolopyridines 15 (Method A)**. A vial was charged, under air, with dithiomalondianilide **12** (300 mg, 1.047 mmol), 3-aryl-2-cyanoacrylate **14** (1.1 mmol) and EtOH (6–8 mL). A mixture was treated with morpholine (0.14 mL, 1.57 mmol) at 25 °C. Complete dissolution of starting materials and formation of a deep-yellow solution occurred for a very short time (a matter of minutes). The solution was stirred for 3 h and left to stand to allow slow evaporation in air at ambient temperature. After evaporating the solvent, the resulting tarry residue was triturated with an appropriate solvent (usually acetone : EtOH (1:1) or n-BuOH were used). The yellow or light-brown crystalline solid was filtered off, washed with EtOH and hexane and recrystallized from acetone (if appropriate) to give pure dithiolopyridines **15**.

**General procedure for the preparation of dithiolopyridines 15 (Method B)**. To a solution of ethyl(methyl) cyanoacetate (1.1 mmol) and an aromatic aldehyde (1.1 mmol) in EtOH(MeOH) (2–3 mL), morpholine (2 drops) was added. Under vigorous stirring, a mixture was warmed up to 50 °C and left to cool to generate 3-aryl-2-cyanoacrylate **14** in situ. Then, 300 mg (1.05 mmol) of dithiomalondianilide **12** and 0.13 mL of morpholine were added. The solution formed was treated as above. The crystals of dithiolopyridine were filtered off, washed with EtOH and hexane and recrystallized (if appropriate).

*Ethyl 6-amino-4-(4-methoxyphenyl)-7-phenyl-3-(phenylimino)-4,7-dihydro-3H-[1,2]di- thiolo[3,4-b]pyridine-5-carboxylate* (**15a**). This compound was prepared via **Method A** in a yield of 388 mg (72%). Alternatively, this compound was prepared via **Method B** employing ethyl cyanoacetate (0.12 mL), anisaldehyde (0.13 mL) and thioamide **12** (300 mg) in a yield of 351 mg (65%), bright yellow crystals. FTIR, ν_max_, cm^−1^: 3400, 3273 (N-H); 1651 (CO_2_Et); 1628 (C=N). ^1^H NMR (400 MHz, DMSO-*d*_6_): 1.09 (t, ^3^*J* = 7.1 Hz, 3H, CH_3_CH_2_), 3.71 (s, 3H, MeO), 3.89–4.03 (m, 2H, CH_3_CH_2_), 5.02 (s, 1H, H-4), 6.84–6.86 (m, 4H, H Ar), 7.00 (very br s, 2H, NH_2_), 7.05–7.09 (m, 1H, H-4 Ph), 7.31–7.34 (m, 4H, H Ar), 7.60–7.66 (m, 5H, H Ar). ^13^C DEPTQ NMR (101 MHz, DMSO-*d*_6_): 14.4* (CH_3_), 38.0* (C-4), 54.9* (OCH_3_), 58.6 (OCH_2_), 78.4 (C-5), 113.2* (2C, CH Ar), 113.5 (C-3a), 120.1* (2C, C2 C-6 Ph), 124.3* (C-4 Ph), 128.5* (2C, CH Ar), 129.7* (2C, C-3 C-5 Ph), 130.0* (2C, CH Ph), 130.5* (2C, CH Ph), 131.1* (C-4 Ph), 135.6 (C-1 Ph), 138.9 (C-1 Ar), 150.9 (C-1 Ph), 152.7 (C-6), 153.7 (C-8a), 157.6 (C–OMe), 163.3 (C=N), 168.7 (C=O). *Signals with a negative phase. Elemental Analysis (C_28_H_25_N_3_O_3_S_2,_ M 515,65): calculated (%): C, 65.22; H, 4.89; N, 8.15; found (%): C, 65.15; H, 5.00; N, 8.12. HRMS (ESI) m/z: calculated for C_28_H_26_N_3_O_3_S_2_ [M + H]^+^: 516.14156, found 516.1411 (Δ 0.89 ppm).

### 3.1. X-ray Studies for Single Crystals of **15a**

Single crystals of 6-amino-4-(4-methoxyphenyl)-7-phenyl-3-(phenylimino)-4,7- dihydro-3H-[1,2]dithiolo[3,4-b]pyridine-5-carboxylate **15a** (C_28_H_25_N_3_O_3_S_2_, M 515.63) were grown from EtOH–acetone solution. The crystal was kept at 423.15(10) K during data collection. Using Olex2 [47], the structure was solved with the olex2.solve structure solution program using Charge Flipping and refined with the SHELXL [48] package using Gauss–Newton minimization. The crystals are orthorhombic, at 423.15(10) K: *a* = 10.2422(4) Å, *b* = 12.9088(5) Å, *c* = 19.2052(7) Å, α = 90°, β = 90°, γ = 90°, *V* = 2539.21(17) Å^3^, *T* = 423.15(10), space group P2_1_2_1_2_1_ (no. 19), Z = 4, *μ*(Cu K_α_) = 2.190 mm^−1^, *d*_calc_ = 1.349 mg/mm^3^, F(000) = 1080.0; a total of 21,592 reflections measured, 4994 unique (*R*_int_ = 0.0510), which were used in all calculations. The final wR_2_ was 0.1035 (all data) and R_1_ was 0.0379 (*I* >2σ(*I*)). A full set of crystallographic data has been deposited in the Cambridge Crystallographic Data Center (CCDC **2219352**).

*Ethyl 6-amino-4-(4-nitrophenyl)-7-phenyl-3-(phenylimino)-4,7-dihydro-3H-[1,2]dithiolo- [3,4-b]pyridine-5-carboxylate* (**15b**). This compound was prepared via **Method A** in a yield of 344 mg (62%), bright-yellow powder. Alternatively, this compound was prepared via **Method B** employing ethyl cyanoacetate (0.11 mL), 4-nitrobenzaldehyde (160 mg) and thioamide **12** (300 mg) in a yield of 240 mg (43%). FTIR, ν_max_, cm^−1^: 3445, 3263 (N-H); 1661 (CO_2_Et); 1630 (C=N); 1524 (NO_2 asym_); 1346 (NO_2 sym_). ^1^H NMR (400 MHz, DMSO-*d*_6_): 0.92 (t, ^3^*J* = 7.0 Hz, 3H, CH_3_CH_2_), 3.81–3.95 (m, 2H, CH_3_CH_2_), 5.94 (s, 1H, H-4), 6.72 (d, ^3^*J* = 7.6 Hz, 2H, H-2 H-6 Ph), 7.02–7.06 (m, 1H, H-4 Ph), 7.19 (very br s, 2H, NH_2_), 7.26–7.30 (m, 2H, H-3 H-5 Ph), 7.38–7.42 (m, 1H, H-4 Ph), 7.65–7.80 (m, 8H, H Ar). ^13^C DEPTQ NMR (101 MHz, DMSO-*d*_6_): 14.1* (CH_3_), 34.6* (C-4), 58.5 (OCH_2_), 76.9 (C-5), 111.7 (C-3a), 120.0* (2C, C2 C-6 Ph), 123.6* (CH Ar), 124.2* (C-4 Ar), 127.2* (CH Ar), 129.4* (2C, C-3 C-5 Ar), 130.3* (2C, CH Ph), 130.5* (2C, CH Ph), 131.3* (C-4 Ph), 133.0* (CH Ar), 135.2 (C-1 Ph), 141.1 (C-1 Ar), 148.8 (C–NO_2_), 150.6 (C-1 Ph), 153.1 (C-6), 154.6 (C-8a), 163.0 (C=N), 168.7 (C=O). *Signals with a negative phase. Elemental Analysis (C_27_H_22_N_4_O_4_S_2,_ M 530,62): calculated (%): C, 61.12; H, 4.18; N, 10.56; found (%): C, 61.03; H, 4.31; N, 10.41. HRMS (ESI) m/z: calculated for C_27_H_23_N_4_O_4_S_2_ [M + H]^+^: 531.11607, found 531.1169 (Δ −1.56 ppm).

*Ethyl 6-amino-7-phenyl-3-(phenylimino)-4-(2-thienyl)-4,7-dihydro-3H-[1,2]dithiolo[3,4- b]pyridine-5-carboxylate* (**15c**). This compound was prepared via **Method A** in a yield of 201 mg (39%). Alternatively, this compound was prepared via **Method B** employing ethyl cyanoacetate (0.11 mL), thiophene-2-carbaldehyde (0.10 mL) and thioamide **12** (300 mg) in a yield of 120 mg (23%), light brown powder. FTIR, ν_max_, cm^−1^: 3380, 3269 (N-H); 1653 (CO_2_Et); 1630 (C=N). ^1^H NMR (400 MHz, DMSO-*d*_6_): 1.12 (t, ^3^*J* = 7.1 Hz, 3H, CH_3_CH_2_), 4.05 (q, ^3^*J* = 7.1 Hz, 2H, CH_3_CH_2_), 5.39 (s, 1H, H-4), 6.92–6.95 (m, 4H, H-3 H-4 thienyl and H-2 H-6 Ph overlapped), 7.09–7.12 (m, 3H, H-4 Ph and NH_2_ overlapped), 7.28 (dd, ^3^*J* = 4.9 Hz, ^4^*J* = 1.5 Hz, 1H, H-5 2-thienyl), 7.34–7.38 (m, 2H, H-3 H-5 Ph), 7.51–7.54 (m, 2H, H-3 H-5 Ph), 7.63–7.65 (m, 3H, H-2 H-6 H-4 Ph). ^13^C DEPTQ NMR (101 MHz, DMSO-*d*_6_): 14.5* (CH_3_), 33.5* (C-4), 58.7 (OCH_2_), 78.0 (C-5), 112.7 (C-3a), 120.1* (2C, C2 C-6 Ph), 122.8* (C-2 thienyl), 123.6* (C-5 thienyl), 124.4* (C-4 Ar), 126.7* (C-4 thienyl), 129.7* (2C, C-3 C-5 Ph), 129.9* (2C, C-3 C-5 Ph), 130.6* (2C, C-2 C-6 Ph), 131.1* (C-4 Ph), 135.5 (C-1 Ph), 150.9 (C-1 Ph), 151.1 (C-1 thienyl), 153.1 (C-6), 154.2 (C-8a), 163.4 (C=N), 168.5 (C=O). *Signals with a negative phase. Elemental Analysis (C_25_H_21_N_3_O_2_S_3,_ M 491,65): calculated (%): C, 61.07; H, 4.31; N, 8.55; found (%): C, 60.98; H, 4.45; N, 8.45. HRMS (ESI) m/z: calculated for C_25_H_22_N_3_O_2_S_3_ [M + H]^+^: 492.0874, found 492.0868 (Δ 1.22 ppm).

*Ethyl 6-amino-4-(4-chlorophenyl)-7-phenyl-3-(phenylimino)-4,7-dihydro-3H-[1,2]dithiolo- [3,4-b]pyridine-5-carboxylate* (**15d**). This compound was prepared via **Method A** in a yield of 300 mg (55%). Alternatively, this compound was prepared via **Method B** employing ethyl cyanoacetate (0.11 mL), 4-chlorobenzaldehyde (150 mg) and thioamide **12** (300 mg) in a yield of 240 mg (46%), yellow powder. FTIR, ν_max_, cm^−1^: 3371, 3271 (N-H); 1655 (CO_2_Et); 1638 (C=N). ^1^H NMR (400 MHz, DMSO-*d*_6_): 1.07 (t, ^3^*J* = 7.0 Hz, 3H, CH_3_CH_2_), 3.96 (q, ^3^*J* = 7.0 Hz, 2H, CH_3_CH_2_), 5.04 (s, 1H, H-4), 6.83 (d, ^3^*J* = 7.6 Hz, 2H, H-2 H-6 Ph), 7.05–7.09 (m, 3H, H-4 Ph and NH_2_ overlapped), 7.30–7.35 (m, 4H, H Ar), 7.41 (d, ^3^*J* = 8.4 Hz, 2H, H Ar), 7.50–7.55 (m, 1H, H-4 Ph), 7.64–7.67 (m, 4H, H Ar). ^13^C DEPTQ NMR (101 MHz, DMSO-*d*_6_): 14.3* (CH_3_), 38.6* (C-4), 58.7 (OCH_2_), 77.7 (C-5), 112.6 (C-3a), 120.1* (2C, C2 C-6 Ph), 124.3* (C-4 Ph), 127.8* (2C, CH Ar), 129.5* (2C, CH Ar), 129.7* (2C, C-3 C-5 Ph), 130.1* (2C, CH Ph), 130.5* (2C, CH Ph), 131.2* (C-4 Ph), 135.4 (C-1 Ph and C-Cl overlapped), 145.6 (C-1 Ar), 150.8 (C-1 Ph), 152.8 (C-6), 154.2 (C-8a), 163.2 (C=N), 168.6 (C=O). *Signals with a negative phase. Elemental Analysis (C_27_H_22_ClN_3_O_2_S_2_ M 520,07): calculated (%): C, 62.36; H, 4.26; N, 8.08; found (%): C, 62.30; H, 4.41; N, 8.05. HRMS (ESI) m/z: calculated for C_27_H_23_ClN_3_O_2_S_2_ [M + H]^+^: 520.09202, found 520.0913 (Δ 1.38 ppm).

*Ethyl 6-amino-4-(2,4-dichlorophenyl)-7-phenyl-3-(phenylimino)-4,7-dihydro-3H-[1,2]- dithiolo[3,4-b]pyridine-5-carboxylate* (**15e**). This compound was prepared via **Method A** in a yield of 215 mg (37%). Alternatively, this compound was prepared via **Method B** employing ethyl cyanoacetate (0.11 mL), 2,4-dichlorobenzaldehyde (180 mg) and thioamide **12** (300 mg) in a yield of 175 mg (30%), brownish-yellow powder. FTIR, ν_max_, cm^−1^: 3474, 3290 (N-H); 1657 (CO_2_Et); 1634 (C=N). ^1^H NMR (400 MHz, DMSO-*d*_6_): 1.05 (t, ^3^*J* = 7.0 Hz, 3H, CH_3_CH_2_), 3.87–3.99 (m, 2H, CH_3_CH_2_), 5.36 (s, 1H, H-4), 6.79 (d, ^3^*J* = 8.3 Hz, 2H, H-2 H-6 Ph), 7.02–7.06 (m, 1H, H-4 Ph), 7.14 (very br s, 2H, NH_2_), 7.27–7.31 (m, 2H, H-3 H-5 Ph), 7.35 (dd, ^3^*J* = 8.6 Hz, ^4^*J* = 2.0 Hz, 1H, H-5 Ar), 7.42 (d, ^4^*J* = 2.0 Hz, 1H, H-3 Ar), 7.52 (d, ^3^*J* = 8.6 Hz, 1H, H-6 Ar), 7.55–7.67 (m, 5H, H Ph). ^13^C DEPTQ NMR (101 MHz, DMSO-*d*_6_): 14.3* (CH_3_), 37.9* (C-4), 58.6 (OCH_2_), 76.5 (C-5), 111.1 (C-3a), 120.1* (2C, C-2 C-6 Ph), 124.2* (C-4 Ph), 126.7* (CH Ar), 128.3* (CH Ar), 129.5* (2C, C-3 C-5 Ph), 130.4* (2C, CH Ph), 130.5* (2C, CH Ph), 130.9 (C–Cl), 131.3* (C-4 Ph), 133.7* (C-3 Ar), 133.8 (C–Cl), 135.3 (C-1 Ph), 142.7 (C-1 Ar), 150.3 (C-1 Ph), 152.9 (C-6), 154.7 (C-8a), 162.2 (C=N), 168.7 (C=O). *Signals with a negative phase. Elemental Analysis (C_27_H_21_Cl_2_N_3_O_2_S_2_ M 554,51): calculated (%):C, 58.48; H, 3.82; N, 7.58; found (%): C, 58.60; H, 3.95; N, 7.41. HRMS (ESI) m/z: calculated for C_27_H_22_Cl_2_N_3_O_2_S_2_ [M + H]^+^: 554.05305, found 554.0532 (Δ −0.27 ppm).

*Methyl 6-amino-4-(4-methoxyphenyl)-7-phenyl-3-(phenylimino)-4,7-dihydro-3H-[1,2]di- thiolo[3,4-b]pyridine-5-carboxylate* (**15f**). This compound was prepared via **Method A** in a yield of 330 mg (63%). Alternatively, this compound was prepared via **Method B** employing methyl cyanoacetate (0.09 mL), 4-methoxybenzaldehyde (0.13 mL) and thioamide **12** (300 mg) in MeOH in a yield of 230 mg (44%), bright-yellow powder. FTIR, ν_max_, cm^−1^: 3452, 3281 (N-H); 1661 (CO_2_Me); 1622 (C=N). ^1^H NMR (400 MHz, DMSO-*d*_6_): 3.51 (s, 3H, COOMe), 3.71 (s, 3H, MeO–Ar), 5.04 (s, 1H, H-4), 6.84–6.88 (m, 4H, H Ar), 7.00 (very br s, 2H, NH_2_), 7.06–7.10 (m, 1H, H-4 Ph), 7.30–7.35 (m, 4H, H Ar), 7.55–7.66 (m, 5H, H Ar). ^13^C DEPTQ NMR (101 MHz, DMSO-*d*_6_): 37.7* (C-4), 50.5* (CO_2_CH_3_), 54.9* (ArOCH_3_), 78.1 (C-5), 113.4* (2C, CH Ar), 113.7 (C-3a), 120.1* (2C, C-2 C-6 Ph), 124.3* (C-4 Ph), 128.2* (2C, CH Ar), 129.7* (2C, C-3 C-5 Ph), 130.0* (2C, CH Ph), 130.5* (2C, CH Ph), 131.1* (C-4 Ph), 135.6 (C-1 Ph), 138.8 (C-1 Ar), 150.9 (C-1 Ph), 153.0 (C-6), 153.6 (C-8a), 157.6 (C–OMe), 163.2 (C=N), 169.1 (C=O). *Signals with a negative phase. Elemental Analysis (C_27_H_23_N_3_O_3_S_2,_ M 501,62): calculated (%): C, 64.65; H, 4.62; N, 8.38; found (%): C, 64.47; H, 4.70; N, 8.25. HRMS (ESI) m/z: calculated for C_27_H_24_N_3_O_3_S_2_ [M + H]^+^: 502.12591, found 502.1248 (Δ 2.21 ppm).

*Methyl 6-amino-7-phenyl-3-(phenylimino)-4-(2-thienyl)-4,7-dihydro-3H-[1,2]dithiolo[3,4- b]pyridine-5-carboxylate* (**15g**). This compound was prepared via **Method A** in a yield of 360 mg (72%). Alternatively, this compound was prepared via **Method B** employing methyl cyanoacetate (0.09 mL), thiophene-2-carbaldehyde (0.09 mL) and thioamide **12** (300 mg) in MeOH in a yield of 326 mg (65%), yellow powder. FTIR, ν_max_, cm^−1^: 3440, 3273 (N-H); 1660 (CO_2_Me); 1627 (C=N). ^1^H NMR (400 MHz, DMSO-*d*_6_): 3.55 (s, 3H, COOMe), 5.40 (s, 1H, H-4), 6.91–6.95 (m, 4H, H-3 H-4 thienyl and H-2 H-6 Ph overlapped), 7.10–7.14 (m, 3H, H-4 Ph and NH_2_ overlapped), 7.26 (dd, ^3^*J* = 5.0 Hz, ^4^*J* = 1.5 Hz, 1H, H-5 2-thienyl), 7.32–7.36 (m, 2H, H-3 H-5 Ph), 7.50–7.54 (m, 2H, H-3 H-5 Ph), 7.60–7.66 (m, 3H, H-2 H-6 H-4 Ph). ^13^C DEPTQ NMR (101 MHz, DMSO-*d*_6_): 34.0* (C-4), 51.1* (CO_2_CH_3_), 78.1 (C-5), 112.8 (C-3a), 120.1* (2C, C2 C-6 Ph), 122.7* (C-2 thienyl), 123.7* (C-5 thienyl), 124.3* (C-4 Ar), 126.6* (C-4 thienyl), 129.7* (2C, C-3 C-5 Ph), 130.0* (2C, C-3 C-5 Ph), 130.5* (2C, C-2 C-6 Ph), 131.0* (C-4 Ph), 135.7 (C-1 Ph), 150.8 (C-1 Ph), 151.0 (C-1 thienyl), 153.3 (C-6), 154.1 (C-8a), 163.4 (C=N), 169.0 (C=O). *Signals with a negative phase. Elemental Analysis (C_24_H_19_N_3_O_2_S_3,_ M 477,62): calculated (%): C, 60.35; H, 4.01; N, 8.80; found (%): C, 60.23; H, 4.15; N, 8.75. HRMS (ESI) m/z: calculated for C_24_H_20_N_3_O_2_S_3_ [M + H]^+^: 478.0718, found 478.0722 (Δ −0.84 ppm).

### 3.2. Herbicide-Safening Effect Studies

Germinated sunflower seeds (cv. Master) with 2–4 mm long embryo roots were placed in a solution of 2,4-D (10^–3^% by weight) for 1 h to achieve 40–60% inhibition of hypocotyl growth. After treatment, the seedlings were washed with pure water and placed into a solution of the corresponding compound **15a,c,f** (concentrations 10^–2^, 10^–3^, 10^–4^ or 10^–5^% by weight, “herbicide + antidote” experiments). After 1 h the seedlings were washed with pure water and placed on paper strips (10 × 75 cm, 20 seeds per strip). The strips were rolled and placed into beakers with water (50 cm^3^). The reference group of seedlings (“herbicide” experiments) was kept in 2,4-D solution (10^–3^%) for 1 h and then in water for 1 h. The “control” seedlings were kept in water for 2 h. The temperature of all solutions was maintained at 28 °C. The seedlings were then thermostated for 3 days at 28 °C. Each experiment was performed in triplicate; 20 seeds were used in each experiment. The results are given in Table 1.

### 3.3. Growth-Stimulating Effect Studies

Sunflower seeds were treated with a solution of a test compound at different concentrations (10^–2^ to 10^–5^ by weight) for 1 h. After 1 h, the seeds were spread evenly on strips of filter paper, rolled up, placed in beakers with water and thermostated at 28 °C for 3 days. Then stem and root length were measured, and the data were statistically processed using Student’s *t*-test, *p* = 0.95. Each experiment was carried out in triplicate with 100 seeds. The results are given in Table 3.

## 4. Conclusions

Generally, a new preparative method for synthesis of alkyl 6-amino-4-aryl-7-phenyl- 3-(phenylimino)-4,7-dihydro-3H-[1,2]dithiolo[3,4-b]pyridine-5-carboxylates **15** based on the base-catalyzed cascade reaction of dithiomalondianilide **12** with alkyl 3-aryl-2-cyanoacrylates was developed. Alternatively, the same compounds can also be prepared in a single step starting from aromatic aldehydes, ethyl (or methyl) cyanoacetate and dithiomalondianilide. Related reactions of dithiomalondianilide **12** with other Michael acceptors are currently underway in our laboratory. Some compounds showed a strong antidote effect with respect to herbicide 2,4-D accompanied by moderate growth-regulating activity in the experiments on sunflower seedlings. Thus, compounds **15a,c,f** reduced the negative effect of 2,4-D on sunflower seedling hypocotyls by 34–60% and by 40–55% on sunflower seedling roots in the laboratory experiments and showed an antidote effect of 41.4–51.4% in the field experiments.

## Figures and Tables

**Table 1:** The antidote effects of the most active compounds **15a,c,f** with respect to herbicide 2,4-D.

N	Compound	Organ	Antidote Effect *A* at Different Concentrations, % ^1^			
			10^−2^	10^−3^	10^−4^	10^−5^
1	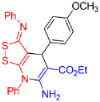 **15a**	roots	146	151	155	151
		hypocotyls	157	160	154	151
2	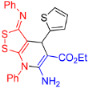 **15c**	roots	140	143	143	140
		hypocotyls	137	134	140	148
3	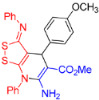 **15f**	roots	133	151	155	151
		hypocotyls	148	154	148	151

^1^ The differences are reliable at *p* = 0.95.

**Table 2:** Results of field tests on the antidote activity of 3H-[1,2]dithiolo[3,4-b]pyridines **15a,c,f** against the herbicide 2,4-D on sunflower cv. Master.

N	Compound	Field Test Variants			
		“Herbicide” (Reference) Group (2,4-D Only)	“Herbicide + Antidote” Group		
		Crop Yield, Quintals per Hectare	Crop Yield, Quintals per Hectare	Antidote Activity	
				Quintals per Hectare	*A_F_*, %
1	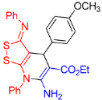 **15a**	14.0	20.8	6.8	48.6 ^1^
2	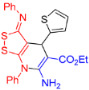 **15c**	14.0	19.8	5.8	41.4 ^1^
3	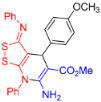 **15f**	14.0	21.2	7.2	51.4 ^1^

^1^ The differences are reliable at *p* = 0.90.

**Table 3:** The growth-stimulating effects of compounds **15a,c,f**.

N	Compound	Organ	Growth-Stimulating Effect *E* at Different Concentrations, % ^1^			
			10^−2^	10^−3^	10^−4^	10^−5^
1	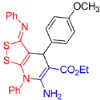 **15a**	roots	116	120	119	118
		stems	115	120	120	122
2	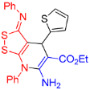 **15c**	roots	108	112	114	112
		stems	114	115	115	116
3	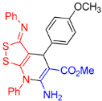 **15f**	roots	112	117	117	118
		stems	113	117	116	112

^1^ The differences are reliable at *p* = 0.95.

## Data Availability

See the file Electronic Supplementary Material.pdf containing ^1^H and ^13^C DEPTQ NMR, 2D NMR ^1^H-^13^C HSQC and ^1^H-^13^C HMBC, FTIR, HRMS spectral charts for dithiolopyridines (Figures S1–S6, S8–S31, Tables S1, S8 and S9) and X-ray crystallography data for dithiolopyridine **15a** (Supplementary Materials Figure S7 and Tables S2–S7).

## Supplementary Materials

The following supporting information can be downloaded at: https://www.mdpi.com/article/10.3390/molecules28020609/s1, Figure S1. FTIR spectrum of dithiolopyridine **15a**; Figure S2. ^1^H NMR spectrum of dithiolopyridine **15a**, DMSO-d_6_ (400 MHz); Figure S3. ^13^C DEPTQ NMR spectrum of dithiolopyridine **15a**, DMSO-d_6_ (101 MHz); Figure S4. ^1^H–^13^C HSQC NMR spectrum of the dithiolopyridine **15**a, DMSO-d_6_ (400/101 MHz); Figure S5. ^1^H–^13^C HMBC NMR spectrum of the dithiolopyridine **15a**, DMSO-d_6_ (400/101 MHz); Figure S6. ^1^H–^13^C HMBC NMR spectrum of the dithiolopyridine 1**5a**, DMSO-d_6_ (400/101 MHz) (fragments); Table S1. The observed correlations in the ^1^H–^13^C HSQC and ^1^H–^13^C HMBC 2D NMR spectra of dithiolopyridine **15a**; Figure S7. ORTEP drawings of the crystal structure showing 50% probability thermal ellipsoids (CCDC 2219352) of the single crystal of compound **15a**; Table S2. Crystal data and structure refinement for dithiolopyridine **15a**; Table S3. Fractional Atomic Coordinates (×10^4^) and Equivalent Isotropic Displacement Parameters (Å^2^×10^3^) for **15a**. U_eq_ is defined as 1/3 of of the trace of the orthogonalised UIJ tensor; Table S4. Anisotropic Displacement Parameters (Å^2^×10^3^) for **15a**. The Anisotropic displacement factor exponent takes the form: -2π^2^[h^2^a*^2^U_11_+...+2hka×b×U_12_]; Table S5. Bond Lengths for dithiolopyridine **15a**; Table S6. Bond Angles for dithiolopyridine **15a**; Table S7. Hydrogen Atom Coordinates (Å×10^4^) and Isotropic Displacement Parameters (Å^2^×10^3^) for **15a**; Figure S8. FTIR spectrum of dithiolopyridine **15b**; Figure S9. ^1^H NMR spectrum of dithiolopyridine **15b**, DMSO-d_6_ (400 MHz); Figure S10. ^13^C DEPTQ NMR spectrum of dithiolopyridine **15b**, DMSO-d_6_ (101 MHz); Figure S11. FTIR spectrum of dithiolopyridine **15c**; Figure S12. ^1^H NMR spectrum of dithiolopyridine **15c**, DMSO-d_6_ (400 MHz); Figure S13. ^13^C DEPTQ NMR spectrum of dithiolopyridine **15c**, DMSO-d_6_ (101 MHz); Figure S14. ^1^H–^13^C HSQC NMR spectrum of the dithiolopyridine **15c**, DMSO-d_6_ (400/101 MHz); Figure S15. ^1^H–^13^C HMBC NMR spectrum of the dithiolopyridine **15c**, DMSO-d_6_ (400/101 MHz); Figure S16. ^1^H–^13^C HMBC NMR spectrum of the dithiolopyridine **15c**, DMSO-d_6_ (400/101 MHz) (fragment); Table S8. The observed correlations in the ^1^H–^13^C HSQC and ^1^H–^13^C HMBC 2D NMR spectra of dithiolopyridine **15c**; Figure S17. FTIR spectrum of dithiolopyridine **15d**; Figure S18. ^1^H NMR spectrum of dithiolopyridine **15d**, DMSO-d_6_ (400 MHz); Figure S19. ^13^C DEPTQ NMR spectrum of dithiolopyridine **15d**, DMSO-d_6_ (101 MHz); Figure S20. FTIR spectrum of dithiolopyridine **15f**; Figure S21. ^1^H NMR spectrum of dithiolopyridine **15f**, DMSO-d_6_ (400 MHz); Figure S22. ^13^C DEPTQ NMR spectrum of dithiolopyridine **15f**, DMSO-d_6_ (101 MHz); Figure S23. ^1^H–^13^C HSQC NMR spectrum of the dithiolopyridine **15f**, DMSO-d_6_ (400/101 MHz); Figure S24. ^1^H–^13^C HMBC NMR spectrum of the dithiolopyridine **15f**, DMSO-d_6_ (400/101 MHz); Figure S25. ^1^H–^13^C HMBC NMR spectrum of the dithiolopyridine **15f**, DMSO-d_6_ (400/101 MHz) (fragment); Table S9. The observed correlations in the ^1^H–^13^C HSQC and ^1^H–^13^C HMBC 2D NMR spectra of dithiolopyridine **15f**; Figure S26. HRMS spectrum of dithiolopyridine **15a**; Figure S27. HRMS spectrum of dithiolopyridine **15b**; Figure S28. HRMS spectrum of dithiolopyridine **15c**; Figure S29. HRMS spectrum of dithiolopyridine **15d**; Figure S30. HRMS spectrum of dithiolopyridine **15e**; Figure S31. HRMS spectrum of dithiolopyridine **15f**.
