# Supplementary material for: Alkyl 4-Aryl-6-amino-7- phenyl-3-(phenylimino)-4,7-dihydro- 3H-[1,2]dithiolo[3,4-b]pyridine-5-carboxylates: Synthesis and Agrochemical Studies
Source: Molecules. 2023 Jan 6;28(2):609. doi: 10.3390/molecules28020609 (PMC9864349; doi:10.3390/molecules28020609)

Electronic Supplementary Material file

Alkyl 4-aryl-6-amino-7- phenyl-3-(phenylimino)-4,7-dihydro-3H-[1,2]dithiolo[3,4-b]pyridine-5-carboxylates: Synthesis and Agrochemical Studies

Victor V. Dotsenko [1,2,*], Anna E. Sinotsko [1], Vladimir D. Strelkov [1], Ekaterina A. Varzieva [1], Alena A. Russkikh [1], Arina G. Levchenko [1], Azamat Z. Temerdashev [3], Nicolai A. Aksenov [2] and Inna V. Aksenova [2]

[1] Department of Organic Chemistry and Technologies, Kuban State University, 350040 Krasnodar, Russia

[2] Department of Chemistry, North Caucasus Federal University, 355017 Stavropol, Russia

[3] Department of Analytical Chemistry, Kuban State University, 350040 Krasnodar, Russia

*Correspondence: victor_dotsenko_@mail.ru (V.V.D.).

Contents. Selected spectral and X-Ray data

Figure S1. FTIR spectrum of dithiolopyridine 15a 3
Figure S2. ^{1}H NMR spectrum of dithiolopyridine 15a, DMSO-d$_6$ (400 MHz) 3
Figure S3. ^{13}C DEPTQ NMR spectrum of dithiolopyridine 15a, DMSO-d$_6$ (101 MHz) 4
Figure S4. ^{1}H–^{13}C HSQC NMR spectrum of the dithiolopyridine 15a, DMSO-d$_6$ (400/101 MHz) 4
Figure S5. ^{1}H–^{13}C HMBC NMR spectrum of the dithiolopyridine 15a, DMSO-d$_6$ (400/101 MHz) 5
Figure S6. ^{1}H–^{13}C HMBC NMR spectrum of the dithiolopyridine 15a, DMSO-d$_6$ (400/101 MHz) (*fragments*) 6
Table S1. The observed correlations in the ^{1}H-^{13}C HSQC and ^{1}H-^{13}C HMBC 2D NMR spectra of dithiolopyridine 15a 7
Figure S7. ORTEP drawings of the crystal structure showing 50% probability thermal ellipsoids (CCDC 2219352) of the single crystal of compound 15a. 8
Table S2. Crystal data and structure refinement for dithiolopyridine 15a 8
Table S3. Fractional Atomic Coordinates (×10^4) and Equivalent Isotropic Displacement Parameters (Å^2×10^3) for 15a. U_{eq} is defined as 1/3 of of the trace of the orthogonalised U_{IJ} tensor 9
Table S4. Anisotropic Displacement Parameters (Å^2×10^3) for 15a. The Anisotropic displacement factor exponent takes the form: $-2\pi^2[h^2a^{*2}U_{11}+...+2hka\times b\times U_{12}]$ 10
Table S5. Bond Lengths for dithiolopyridine 15a. 11
Table S6. Bond Angles for dithiolopyridine 15a. 11
Table S7. Hydrogen Atom Coordinates (Å×10^4) and Isotropic Displacement Parameters (Å^2×10^3) for 15a. 12

Figure S8. FTIR spectrum of dithiolopyridine 15b 13
Figure S9. ^{1}H NMR spectrum of dithiolopyridine 15b, DMSO-d_6 (400 MHz) 13
Figure S10. ^{13}C DEPTQ NMR spectrum of dithiolopyridine 15b, DMSO-d_6 (101 MHz) 14
Figure S11. FTIR spectrum of dithiolopyridine 15c 14
Figure S12. ^{1}H NMR spectrum of dithiolopyridine 15c, DMSO-d_6 (400 MHz) 15
Figure S13. ^{13}C DEPTQ NMR spectrum of dithiolopyridine 15c, DMSO-d_6 (101 MHz) 15
Figure S14. ^{1}H–^{13}C HSQC NMR spectrum of the dithiolopyridine 15c, DMSO-d_6 (400/101 MHz) 16
Figure S15. ^{1}H–^{13}C HMBC NMR spectrum of the dithiolopyridine 15c, DMSO-d_6 (400/101 MHz) 17
Figure S16. ^{1}H–^{13}C HMBC NMR spectrum of the dithiolopyridine 15c, DMSO-d_6 (400/101 MHz) (fragment) 18
Table S8. The observed correlations in the ^{1}H-^{13}C HSQC and ^{1}H-^{13}C HMBC 2D NMR spectra of dithiolopyridine 15c 19
Figure S17. FTIR spectrum of dithiolopyridine 15d 20
Figure S18. ^{1}H NMR spectrum of dithiolopyridine 15d, DMSO-d_6 (400 MHz) 20
Figure S19. ^{13}C DEPTQ NMR spectrum of dithiolopyridine 15d, DMSO-d_6 (101 MHz) 21
Figure S20. FTIR spectrum of dithiolopyridine 15f 21
Figure S21. ^{1}H NMR spectrum of dithiolopyridine 15f, DMSO-d_6 (400 MHz) 22
Figure S22. ^{13}C DEPTQ NMR spectrum of dithiolopyridine 15f, DMSO-d_6 (101 MHz) 22
Figure S23. ^{1}H–^{13}C HSQC NMR spectrum of the dithiolopyridine 15f, DMSO-d_6 (400/101 MHz) 23
Figure S24. ^{1}H–^{13}C HMBC NMR spectrum of the dithiolopyridine 15f, DMSO-d_6 (400/101 MHz) 24
Figure S25. ^{1}H–^{13}C HMBC NMR spectrum of the dithiolopyridine 15f, DMSO-d_6 (400/101 MHz) (*fragment*) 25
Table S9. The observed correlations in the ^{1}H-^{13}C HSQC and ^{1}H-^{13}C HMBC 2D NMR spectra of dithiolopyridine 15f 26
Figure S26. HRMS spectrum of dithiolopyridine 15a 27
Figure S27. HRMS spectrum of dithiolopyridine 15b 27
Figure S28. HRMS spectrum of dithiolopyridine 15c 28
Figure S29. HRMS spectrum of dithiolopyridine 15d 28
Figure S30. HRMS spectrum of dithiolopyridine 15e 29
Figure S31. HRMS spectrum of dithiolopyridine 15f 29

Figure S1. FTIR spectrum of dithiolopyridine 15a

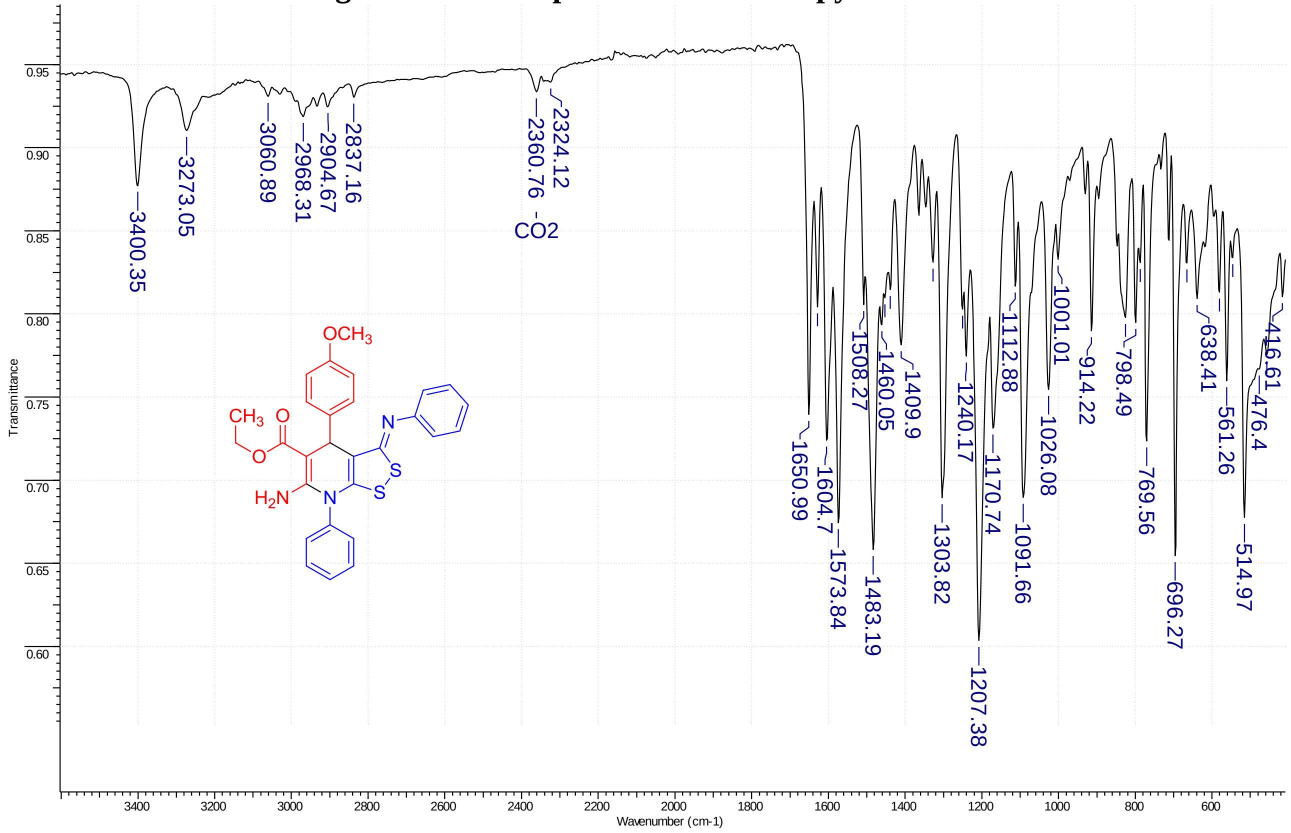

Figure S2. ^{1}H NMR spectrum of dithiolopyridine 15a, DMSO-d_6 (400 MHz)

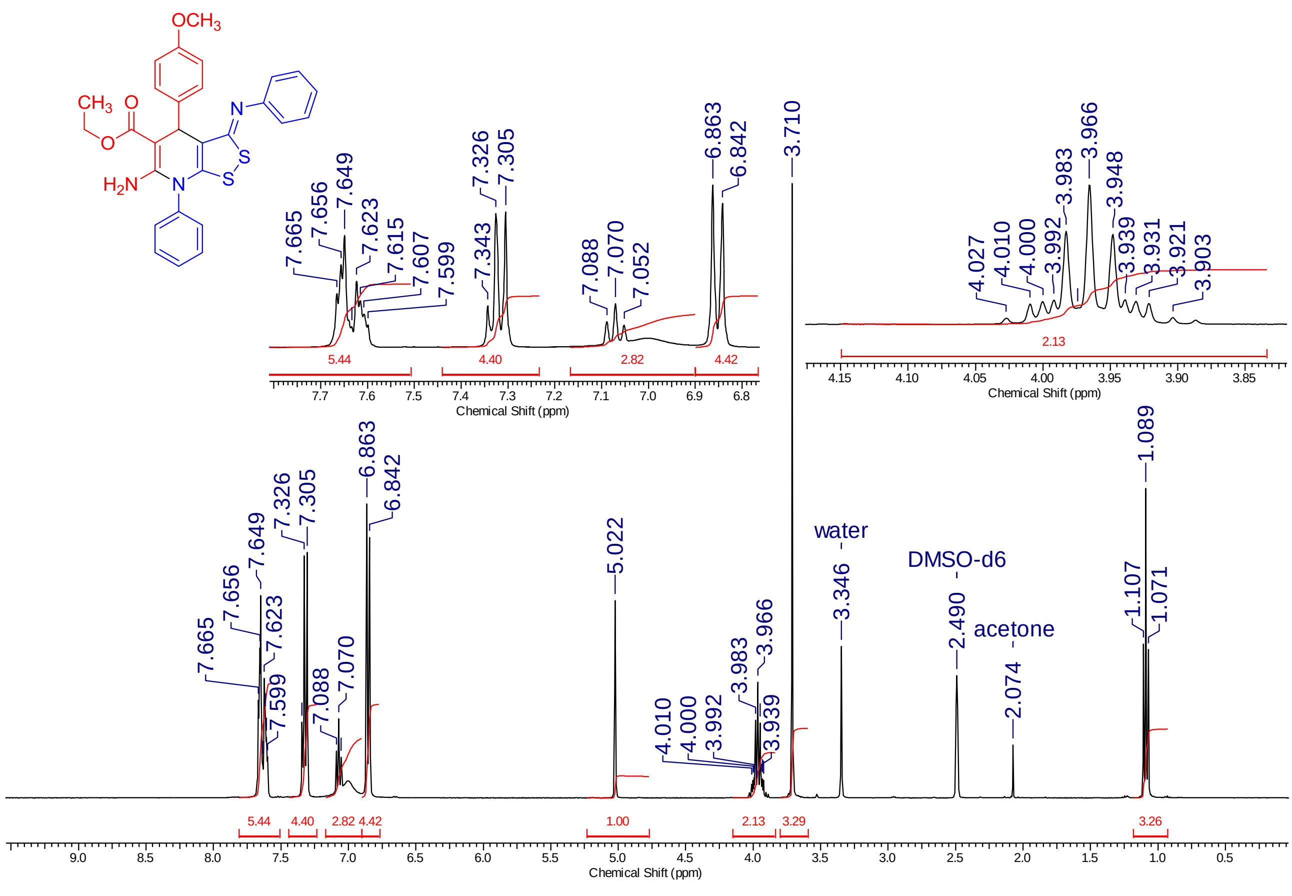

Figure S3. ^{13}C DEPTQ NMR spectrum of dithiolopyridine 15a, DMSO-d_6 (101 MHz)

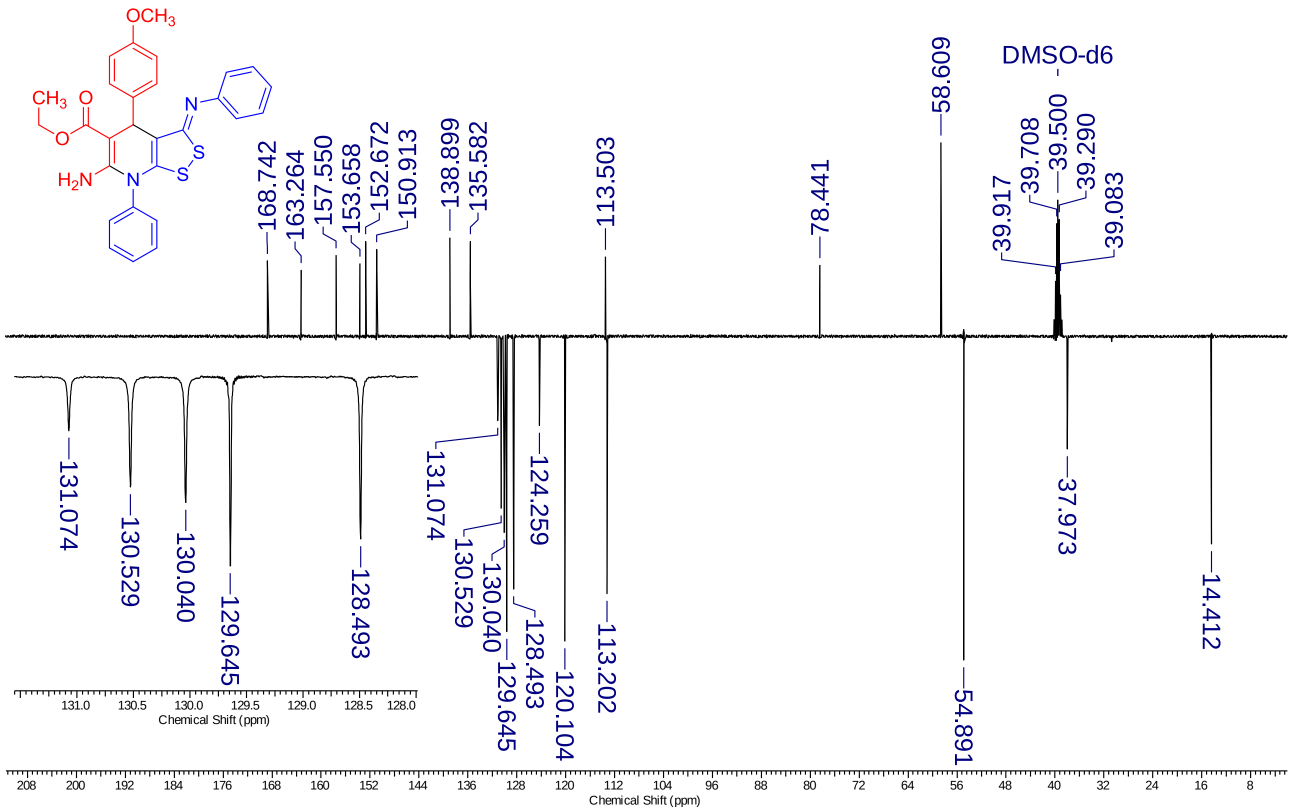

Figure S4. ^{1}H–^{13}C HSQC NMR spectrum of the dithiolopyridine 15a, DMSO-d_6 (400/101 MHz)

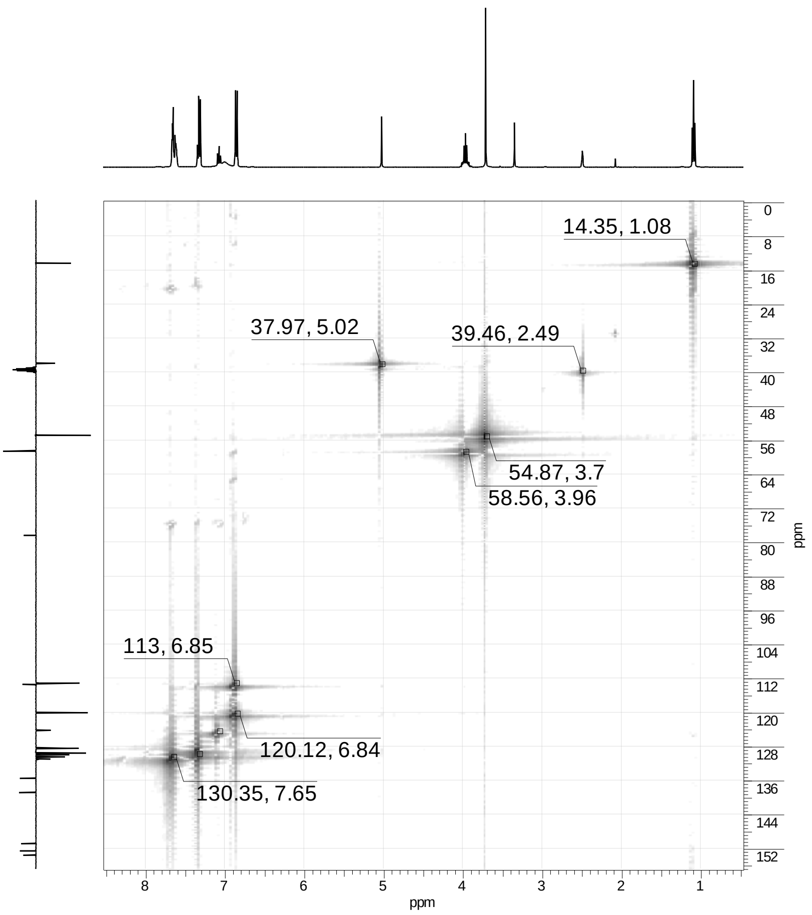

Figure S5. ^{1}H–^{13}C HMBC NMR spectrum of the dithiolopyridine 15a, DMSO-d_6 (400/101 MHz)

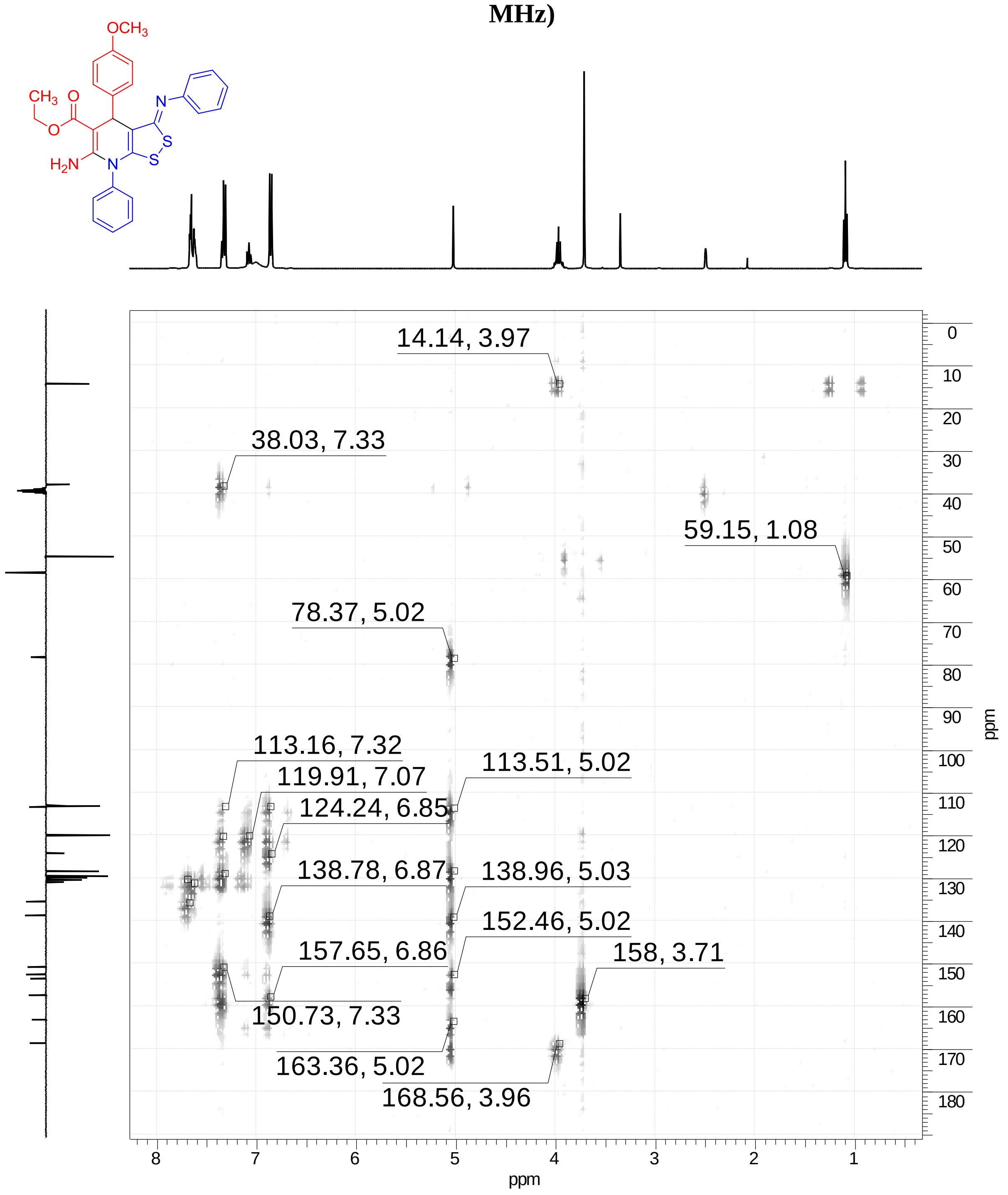

Figure S6. ^{1}H–^{13}C **HMBC NMR spectrum of the dithiolopyridine 15a, DMSO-d_6 (400/101 MHz) (*fragments*)**

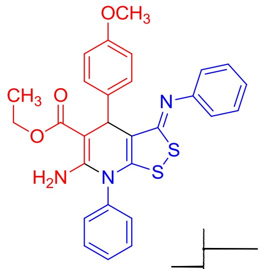

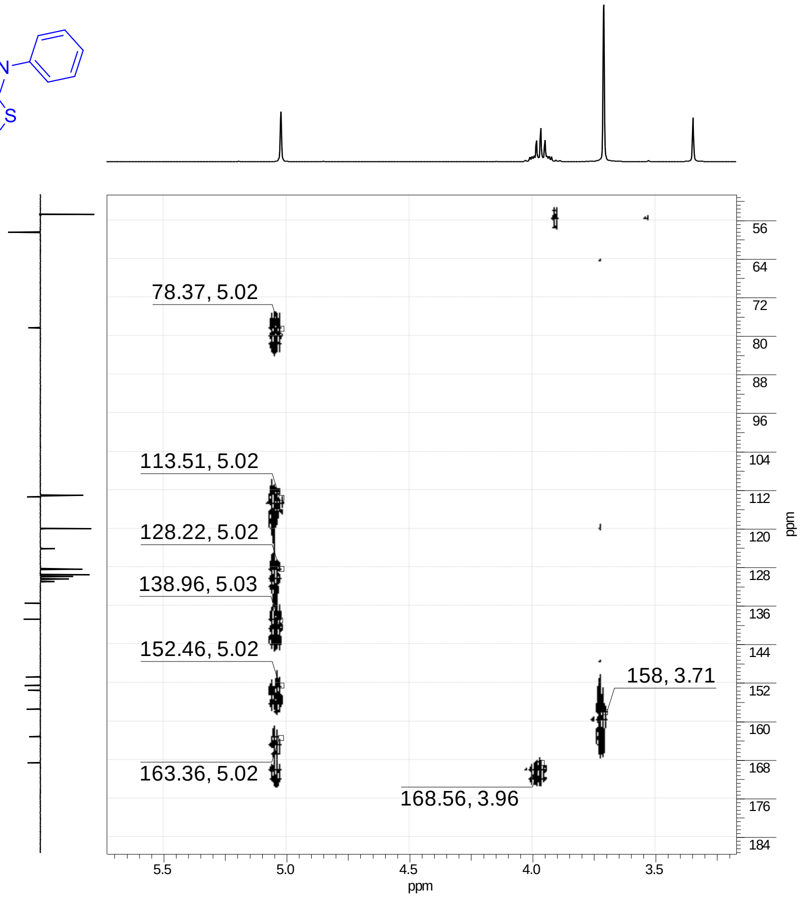

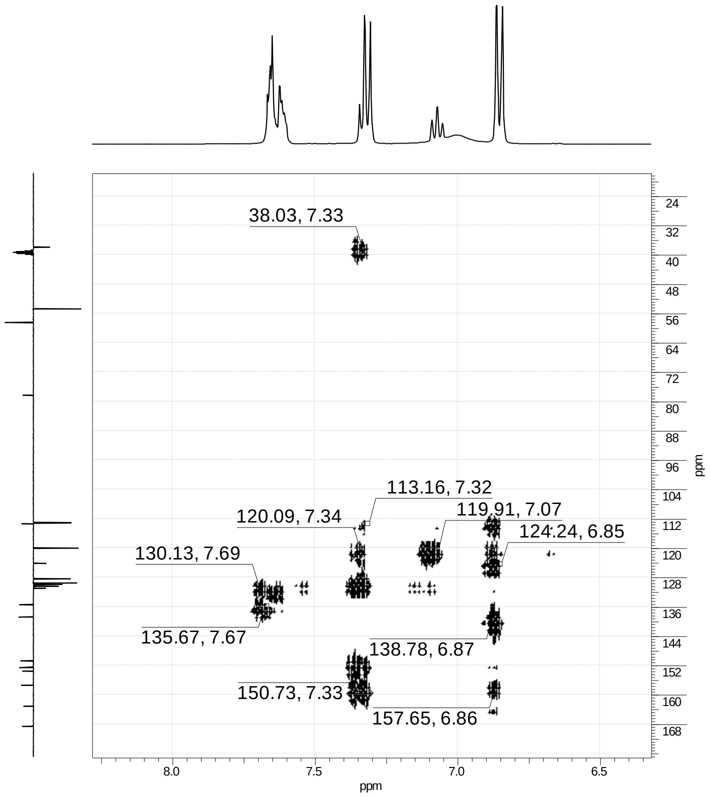

Table S1. The observed correlations in the ^{1}H-^{13}C HSQC and ^{1}H-^{13}C HMBC 2D NMR spectra of dithiolopyridine 15a

^{13}C chemical shifts are given in **red**, ^{1}H shifts – in **blue**

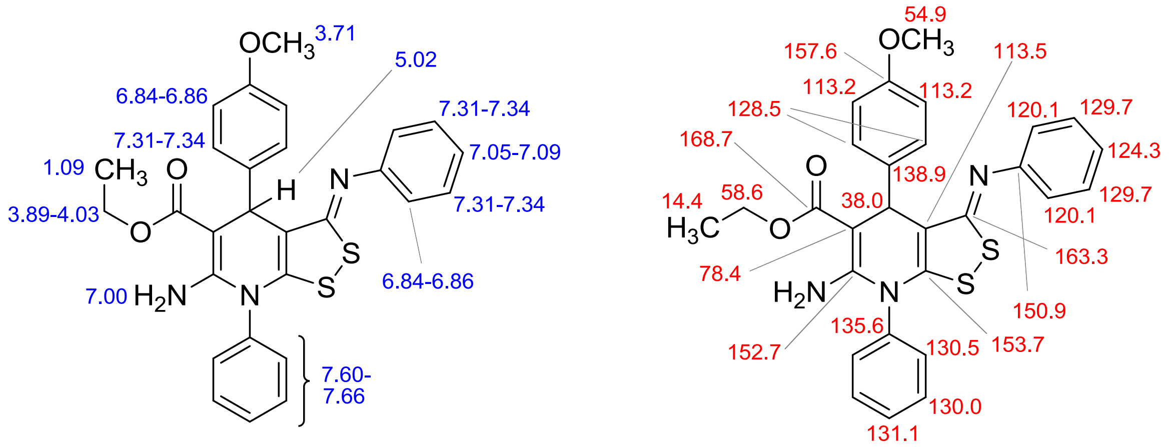

^{1}H NMR shifts, δ, ppm	Correlations in HSQC spectrum, δ, ppm	Correlations in HMBC spectrum, δ, ppm
1.09 (t, ^{3}J = 7.1 Hz, 3H, C<u>H</u>$_3$CH$_2$)	14.4* (CH$_3$)	58.6 (OCH$_2$)
3.71 (s, 3H, MeO)	54.9* (OCH$_3$)	157.6 (C–OMe)
3.89–4.03 (m, 2H, CH$_3$C<u>H</u>$_2$)	58.6 (OCH$_2$)	14.4* (CH$_3$), 168.7 (C=O)
5.02 (s, 1H, H-4)	38.0* (C-4)	78.4 (C-5), 113.5 (C-3a), 128.5* (2C, CH Ar), 138.9 (C-1 Ar), 152.7 (C-6), 153.7 (C-8a), 163.3 (C=N), 168.7 (C=O)
6.84–6.86 (m, 4H, H Ar)	113.2* (2C, CH Ar) 120.1* (2C, CH Ar)	113.2* (2C, CH Ar), 120.1* (2C, CH Ar), 124.3* (CH Ar), 138.9 (C-1 Ar), 157.6 (C–OMe)
7.00 (very br s, 2H, NH$_2$)	–	–
7.05–7.09 (m, 1H, H-4 Ph)	124.3* (CH Ar)	120.1* (2C, CH Ar), 129.7* (2C, CH Ar)
7.31–7.34 (m, 4H, H Ar)	128.5* (2C, CH Ar), 129.7* (2C, CH Ar)	38.0* (C-4), 113.2* (2C, CH Ar), 128.5* (2C, CH Ar), 129.7* (2C, CH Ar), 150.9 (C-1 Ph), 157.6 (C–OMe)
7.60–7.66 (m, 5H, H Ar)	130.0* (CH Ar), 130.5* (CH Ar), 131.1* (CH Ar)	130.0* (CH Ar), 130.5* (CH Ar), 131.1* (CH Ar), 135.6 (C-1 Ph)

*Signals with a negative phase.

Figure S7. ORTEP drawings of the crystal structure showing 50% probability thermal ellipsoids (CCDC 2219352) of the single crystal of compound 15a.

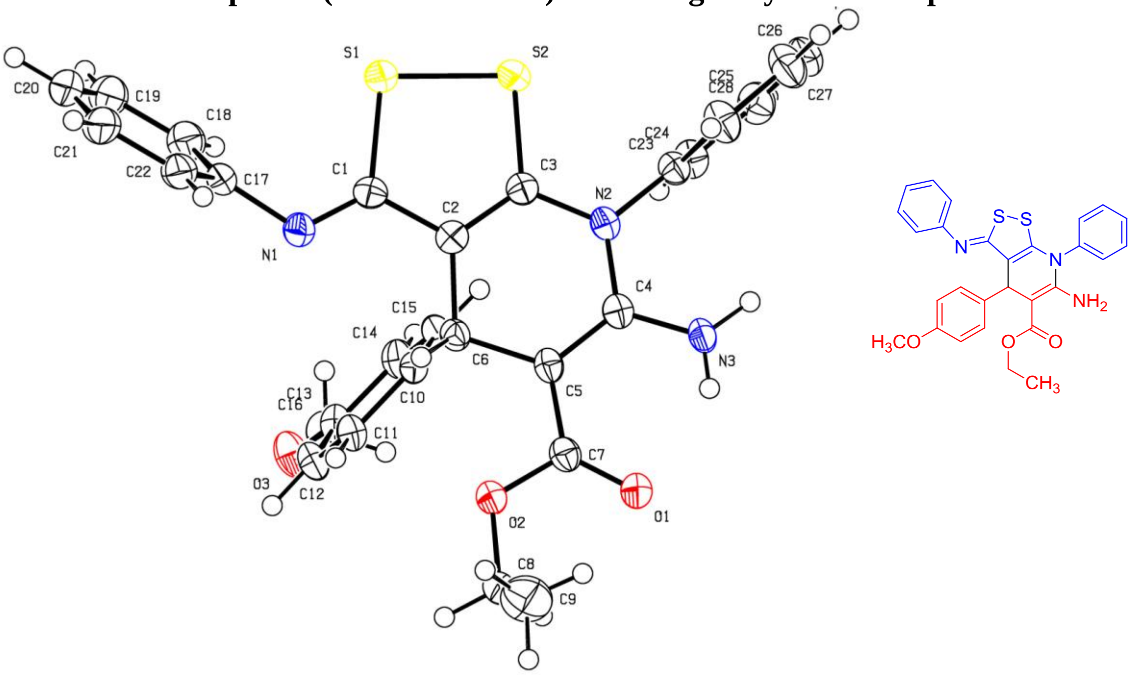

Table S2. Crystal data and structure refinement for dithiolopyridine 15a

Empirical formula	$C_{28}H_{25}N_3O_3S_2$
Formula weight	515.63
Temperature/K	423.15(10)
Crystal system	orthorhombic
Space group	$P2_12_12_1$
a/Å	10.2422(4)
b/Å	12.9088(5)
c/Å	19.2052(7)
α/°	90
β/°	90
γ/°	90
Volume/Å^3	2539.21(17)
Z	4
ρ_{calc}mg/mm^3	1.349
m/mm^{-1}	2.190
F(000)	1080.0
Crystal size/mm^3	0.421 × 0.288 × 0.13
2Θ range for data collection	8.252 to 144.204°
Index ranges	$-11 \leq h \leq 12$, $-15 \leq k \leq 15$, $-23 \leq l \leq 23$
Reflections collected	21592
Independent reflections	4994[R(int) = 0.0510]
Data/restraints/parameters	4994/0/311
Goodness-of-fit on F^2	1.029
Final R indexes [I>=2σ (I)]	R_1 = 0.0379, wR_2 = 0.1016
Final R indexes [all data]	R_1 = 0.0394, wR_2 = 0.1035
Largest diff. peak/hole / e Å^{-3}	0.46/-0.29
Flack parameter	0.010(9)

Table S3. Fractional Atomic Coordinates (×10^4) and Equivalent Isotropic Displacement Parameters (Å^2×10^3) for 15a. U_{eq} is defined as 1/3 of of the trace of the orthogonalised U_{IJ} tensor.

Atom	*x*	*y*	*z*	U(eq)
S2	2865.5(7)	4442.3(5)	3910.5(3)	29.60(18)
S1	4510.7(7)	5224.8(5)	4252.7(3)	29.65(18)
O1	2480(2)	5596.0(16)	499.5(11)	32.1(4)
O2	4361(2)	6358.5(17)	822(1)	33.4(5)
O3	8936(2)	3081.9(16)	1367.1(13)	37.3(5)
N2	2229(2)	4397.6(18)	2556.8(12)	27.4(5)
N3	1219(2)	4541.9(18)	1482.7(14)	27.2(5)
N1	6032(2)	6244.4(18)	3318.2(13)	28.4(5)
C5	3322(3)	5354(2)	1648.2(14)	24.7(5)
C7	3315(3)	5753(2)	950.9(15)	26.9(5)
C12	7815(3)	4662(2)	1378.9(16)	31.4(6)
C6	4522(3)	5537(2)	2106.1(13)	24.3(5)
C15	5693(3)	3799(2)	2044.4(15)	27.9(6)
C10	5682(3)	4858(2)	1896.6(13)	23.4(5)
C11	6758(3)	5272(2)	1557.4(14)	27.9(6)
C4	2280(3)	4768(2)	1874.9(14)	24.6(5)
C14	6750(3)	3177(2)	1876.6(16)	30.6(6)
C17	6848.6(18)	6485.0(14)	3899.1(9)	28.6(6)
C22	6931(2)	7505.1(12)	4126.5(10)	35.2(7)
C21	7765(2)	7762.5(13)	4670.6(10)	45.0(9)
C20	8517(2)	6999.7(18)	4987.2(10)	48.2(9)
C19	8435(2)	5979.6(16)	4759.7(11)	47.5(9)
C18	7600(2)	5722.2(12)	4215.7(11)	39.0(7)
C2	4175(3)	5336(2)	2857.1(14)	25.0(5)
C3	3144(3)	4759(2)	3035.6(14)	24.8(5)
C1	5034(3)	5677(2)	3407.7(14)	25.5(5)
C23	1249.7(16)	3684.9(12)	2781.4(10)	26.5(5)
C24	1490.5(16)	2626.9(13)	2740.1(11)	31.2(6)
C25	562(2)	1925.4(10)	2978.0(12)	38.1(7)
C26	-606.2(18)	2281.8(13)	3257.2(12)	40.7(7)
C27	-847.0(16)	3339.7(15)	3298.5(12)	43.4(8)
C28	80.9(18)	4041.3(10)	3060.7(12)	35.5(7)
C13	7822(3)	3614(2)	1544.9(15)	29.2(6)
C16	8864(3)	1975(2)	1379.7(19)	37.9(7)
C8	4462(3)	6809(3)	134.3(16)	40.7(7)
C9	3610(5)	7752(3)	60(2)	59.4(11)

Table S4. Anisotropic Displacement Parameters ($Å^2×10^3$) for 15a. The Anisotropic displacement factor exponent takes the form: $-2π^2[h^2a^{*2}U_{11}+...+2hka×b×U_{12}]$

Atom	U_{11}	U_{22}	U_{33}	U_{23}	U_{13}	U_{12}
S2	29.4(3)	32.6(3)	26.8(3)	2.0(3)	3.2(3)	-6.0(3)
S1	29.5(4)	34.0(3)	25.4(3)	-1.4(2)	1.0(3)	-4.3(3)
O1	28.3(10)	37.3(11)	30.8(10)	1.8(8)	-2.9(8)	-6.7(8)
O2	26.1(11)	44.2(11)	29.9(10)	7.1(9)	-2.3(8)	-9.2(9)
O3	25.3(10)	30.2(11)	56.4(14)	-5.3(9)	5.3(10)	2.0(8)
N2	24.7(12)	28.4(11)	29.1(11)	0.6(9)	0.6(9)	-6.7(10)
N3	20.2(11)	30.1(12)	31.4(12)	1.9(10)	-0.4(9)	-5.6(9)
N1	27.9(12)	29.3(12)	28.0(11)	-2.2(9)	-0.1(10)	-4.4(10)
C5	20.3(12)	23.7(12)	30.1(13)	-2.8(10)	0.7(10)	-0.3(10)
C7	22.7(13)	27.2(13)	30.8(13)	-1.4(11)	2.0(11)	0.6(10)
C12	23.2(14)	32.0(14)	39.0(15)	-2.1(12)	4.2(12)	-5.6(11)
C6	23.9(12)	22.5(11)	26.3(12)	-0.5(9)	0.7(11)	-3.3(10)
C15	22.7(14)	28.7(13)	32.4(14)	1.6(11)	1.7(11)	-3(1)
C10	21.1(13)	24.2(12)	24.9(12)	-1.4(10)	-1.8(10)	-1.8(10)
C11	24.5(14)	25.8(12)	33.4(14)	-1.1(11)	-0.8(11)	-4.4(11)
C4	22.1(13)	21.4(11)	30.4(13)	-1.7(10)	0.6(10)	2.8(10)
C14	29.2(15)	24.5(12)	38.1(15)	-1.8(11)	-1.3(12)	-1.8(11)
C17	25.2(14)	34.6(14)	26.1(13)	-0.4(11)	1.7(11)	-7.4(11)
C22	40.6(17)	33.9(14)	31.2(14)	-0.7(12)	0.7(13)	-10.9(13)
C21	56(2)	47.1(19)	32.1(15)	-4.1(13)	-0.7(15)	-24.4(17)
C20	44(2)	69(2)	31.2(16)	1.3(15)	-3.9(14)	-23.8(17)
C19	32.7(18)	68(2)	42.3(18)	11.8(16)	-4.2(15)	1.2(16)
C18	34.2(16)	42.0(17)	40.6(16)	0.3(13)	1.0(14)	1.0(13)
C2	26.9(14)	21.4(12)	26.6(13)	-1.2(10)	-0.1(10)	1.7(10)
C3	25.1(13)	23.6(12)	25.8(12)	-2.1(10)	1.2(10)	1(1)
C1	27.5(13)	23.4(12)	25.4(12)	-1.9(10)	2.9(10)	2.3(10)
C23	25.3(14)	25.7(12)	28.4(13)	-0.2(10)	1.6(11)	-2.2(10)
C24	29.0(15)	26.6(13)	38.0(14)	1.2(11)	-1.2(12)	0.8(11)
C25	43.1(18)	26.2(13)	45.0(17)	5.1(12)	-5.5(15)	-7.7(13)
C26	41.0(18)	39.1(16)	42.2(16)	1.4(13)	4.0(15)	-18.6(14)
C27	33.0(18)	45.5(18)	51.7(19)	-10.2(15)	11.8(15)	-7.9(14)
C28	30.6(15)	28.8(14)	47.1(17)	-6.0(13)	6.6(14)	-1.8(11)
C13	23.7(14)	28.5(13)	35.3(14)	-5.5(11)	-2.2(12)	0.2(11)
C16	35.4(17)	30.3(15)	48.0(18)	-4.8(13)	2.6(14)	7.9(13)
C8	32.4(16)	58.7(19)	31.0(14)	11.8(14)	-1.8(13)	-14.9(15)
C9	74(3)	45(2)	59(2)	17.5(18)	-2(2)	-7.6(19)

Table S5. Bond Lengths for dithiolopyridine 15a.

Atom	Atom	Length/Å	Atom	Atom	Length/Å
S2	S1	2.0716(10)	C6	C2	1.508(4)
S2	C3	1.753(3)	C15	C10	1.397(4)
S1	C1	1.806(3)	C15	C14	1.385(4)
O1	C7	1.234(4)	C10	C11	1.387(4)
O2	C7	1.349(3)	C14	C13	1.390(4)
O2	C8	1.447(4)	C17	C22	1.3900
O3	C13	1.374(4)	C17	C18	1.3900
O3	C16	1.431(4)	C22	C21	1.3900
N2	C4	1.395(4)	C21	C20	1.3900
N2	C3	1.393(4)	C20	C19	1.3900
N2	C23	1.428(3)	C19	C18	1.3900
N3	C4	1.354(4)	C2	C3	1.337(4)
N1	C17	1.429(3)	C2	C1	1.444(4)
N1	C1	1.269(4)	C23	C24	1.3900
C5	C7	1.435(4)	C23	C28	1.3900
C5	C6	1.530(4)	C24	C25	1.3900
C5	C4	1.379(4)	C25	C26	1.3900
C12	C11	1.381(4)	C26	C27	1.3900
C12	C13	1.390(4)	C27	C28	1.3900
C6	C10	1.530(4)	C8	C9	1.505(6)

Table S6. Bond Angles for dithiolopyridine 15a.

Atom	Atom	Atom	Angle/°	Atom	Atom	Atom	Angle/°
C3	S2	S1	93.32(10)	C22	C17	C18	120.0
C1	S1	S2	96.55(10)	C18	C17	N1	120.79(16)
C7	O2	C8	117.2(2)	C17	C22	C21	120.0
C13	O3	C16	116.9(2)	C20	C21	C22	120.0
C4	N2	C23	122.0(2)	C19	C20	C21	120.0
C3	N2	C4	118.7(2)	C20	C19	C18	120.0
C3	N2	C23	119.3(2)	C19	C18	C17	120.0
C1	N1	C17	119.4(2)	C3	C2	C6	121.8(2)
C7	C5	C6	119.0(2)	C3	C2	C1	117.6(2)
C4	C5	C7	119.2(3)	C1	C2	C6	120.3(2)
C4	C5	C6	121.7(2)	N2	C3	S2	116.4(2)
O1	C7	O2	121.1(3)	C2	C3	S2	120.3(2)
O1	C7	C5	126.8(3)	C2	C3	N2	123.3(3)
O2	C7	C5	112.1(2)	N1	C1	S1	123.2(2)
C11	C12	C13	120.1(3)	N1	C1	C2	124.6(3)
C10	C6	C5	112.6(2)	C2	C1	S1	112.2(2)
C2	C6	C5	109.5(2)	C24	C23	N2	119.41(15)
C2	C6	C10	109.6(2)	C24	C23	C28	120.0
C14	C15	C10	121.8(3)	C28	C23	N2	120.55(15)
C15	C10	C6	120.9(2)	C25	C24	C23	120.0
C11	C10	C6	121.4(2)	C24	C25	C26	120.0
C11	C10	C15	117.7(3)	C27	C26	C25	120.0
C12	C11	C10	121.3(3)	C28	C27	C26	120.0
N3	C4	N2	114.8(2)	C27	C28	C23	120.0
N3	C4	C5	124.3(3)	O3	C13	C12	115.7(3)

C5	C4	N2	120.9(2)	O3	C13	C14	124.5(3)
C15	C14	C13	119.3(3)	C14	C13	C12	119.7(3)
C22	C17	N1	119.14(15)	O2	C8	C9	111.7(3)

Table S7. Hydrogen Atom Coordinates (Å×10^4) and Isotropic Displacement Parameters (Å^2×10^3) for 15a.

Atom	*x*	*y*	*z*	U(eq)
H12	8522.98	4953.33	1147.15	38
H6	4781.51	6264.39	2060.36	29
H15	4972.41	3502.85	2261.31	34
H11	6768.89	5973.58	1448.02	33
H14	6741.22	2474.85	1984.7	37
H22	6428.02	8015.48	3914.72	42
H21	7820.34	8445.03	4822.78	54
H20	9075.12	7171.93	5351.19	58
H19	8937.59	5469.26	4971.54	57
H18	7545.29	5039.69	4063.48	47
H24	2272.41	2388.49	2553.3	37
H25	723.58	1217.61	2950.3	46
H26	-1227.11	1812.44	3416.31	49
H27	-1628.98	3578.16	3485.33	52
H28	-80.17	4749.07	3088.33	43
H16A	8167.38	1747.53	1082.28	57
H16B	8702.09	1745.04	1847.12	57
H16C	9674.91	1689.71	1218.03	57
H8A	4207.88	6297.59	-210.12	49
H8B	5362.96	6999.27	45.69	49
H9A	3730.66	8048.21	-394.16	89
H9B	3843.09	8252.35	408.27	89
H9C	2711.45	7557.99	117.98	89
H3A	1290(30)	4710(30)	1070(20)	26(8)
H3B	530(40)	4120(30)	1610(20)	47(11)

Figure S8. FTIR spectrum of dithiolopyridine 15b

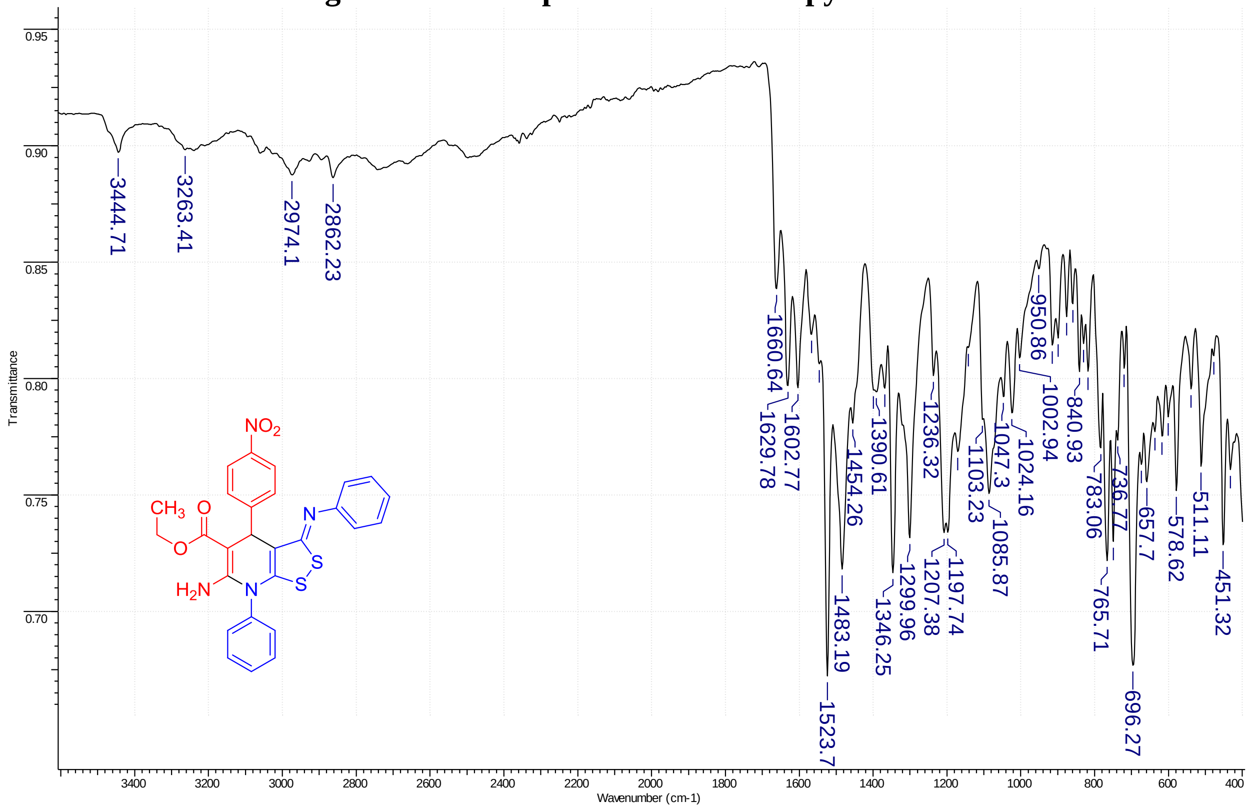

Figure S9. ^{1}H NMR spectrum of dithiolopyridine 15b, DMSO-d_6 (400 MHz)

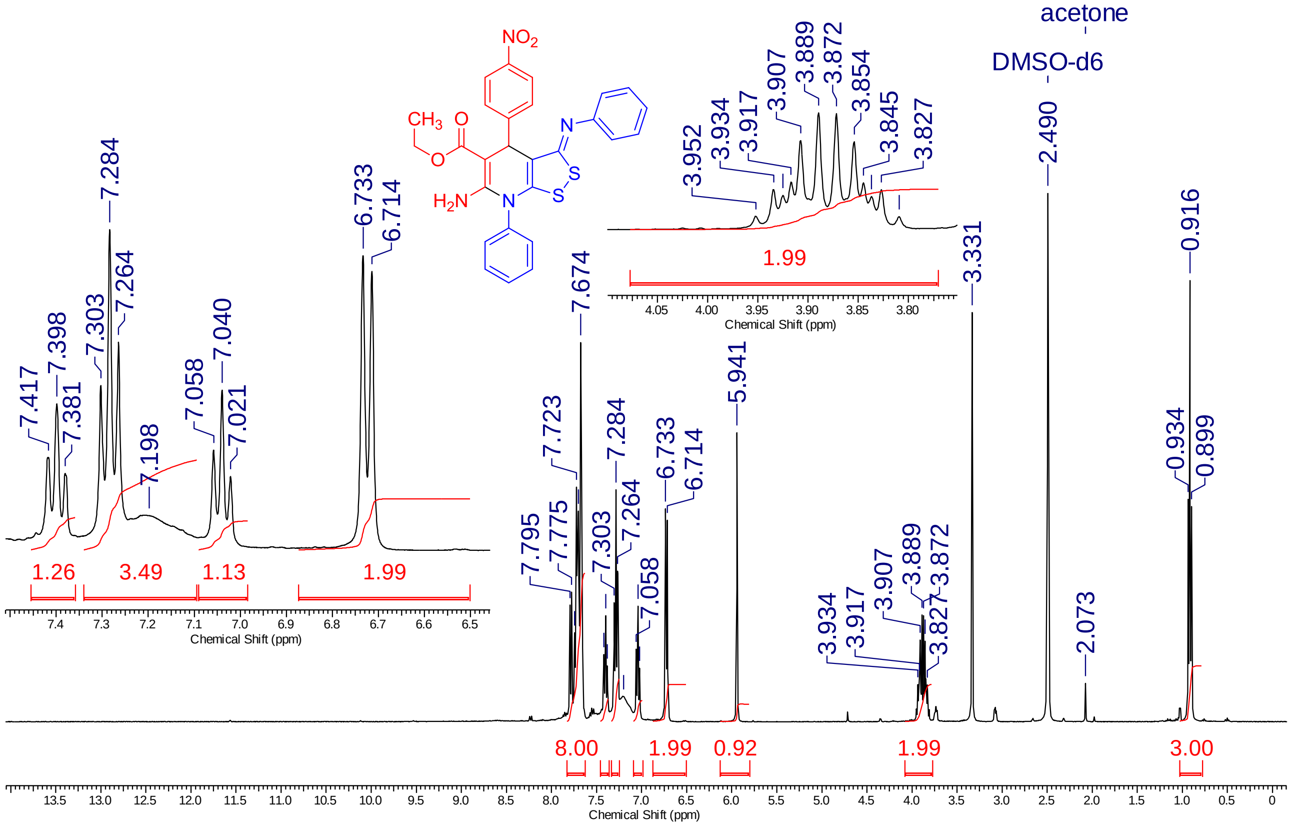

Figure S10. ^{13}C **DEPTQ NMR spectrum of dithiolopyridine 15b, DMSO-d$_6$ (101 MHz)**

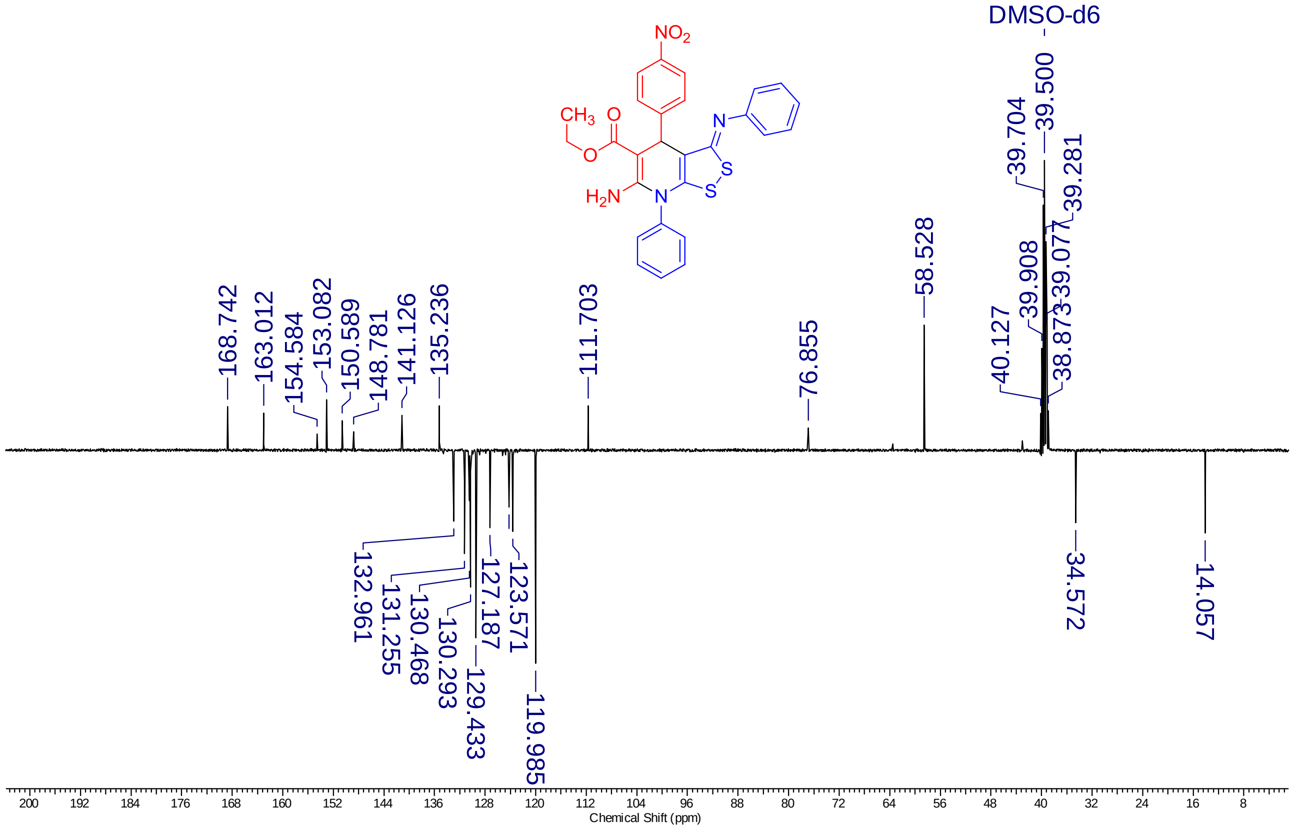

Figure S11. FTIR spectrum of dithiolopyridine 15c

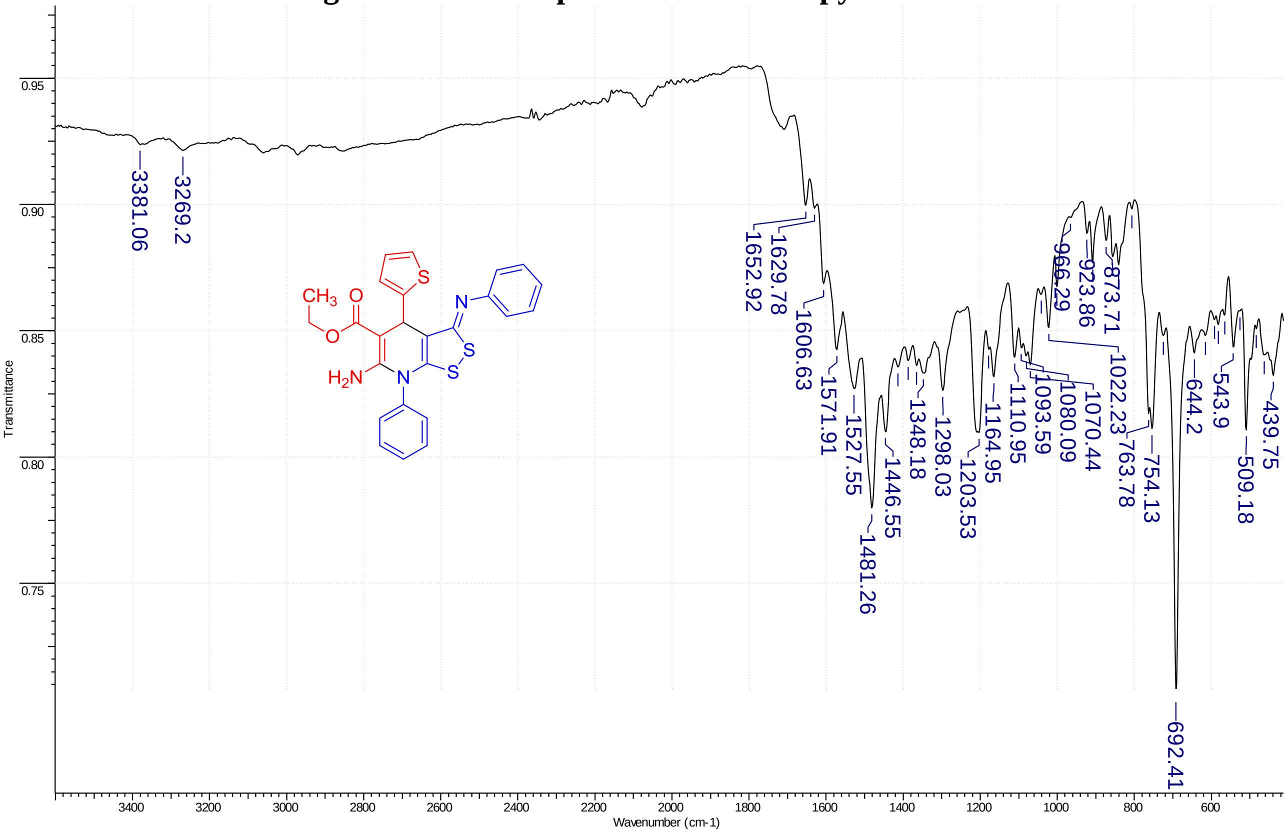

Figure S12. ^{1}H NMR spectrum of dithiolopyridine 15c, DMSO-d$_6$ (400 MHz)

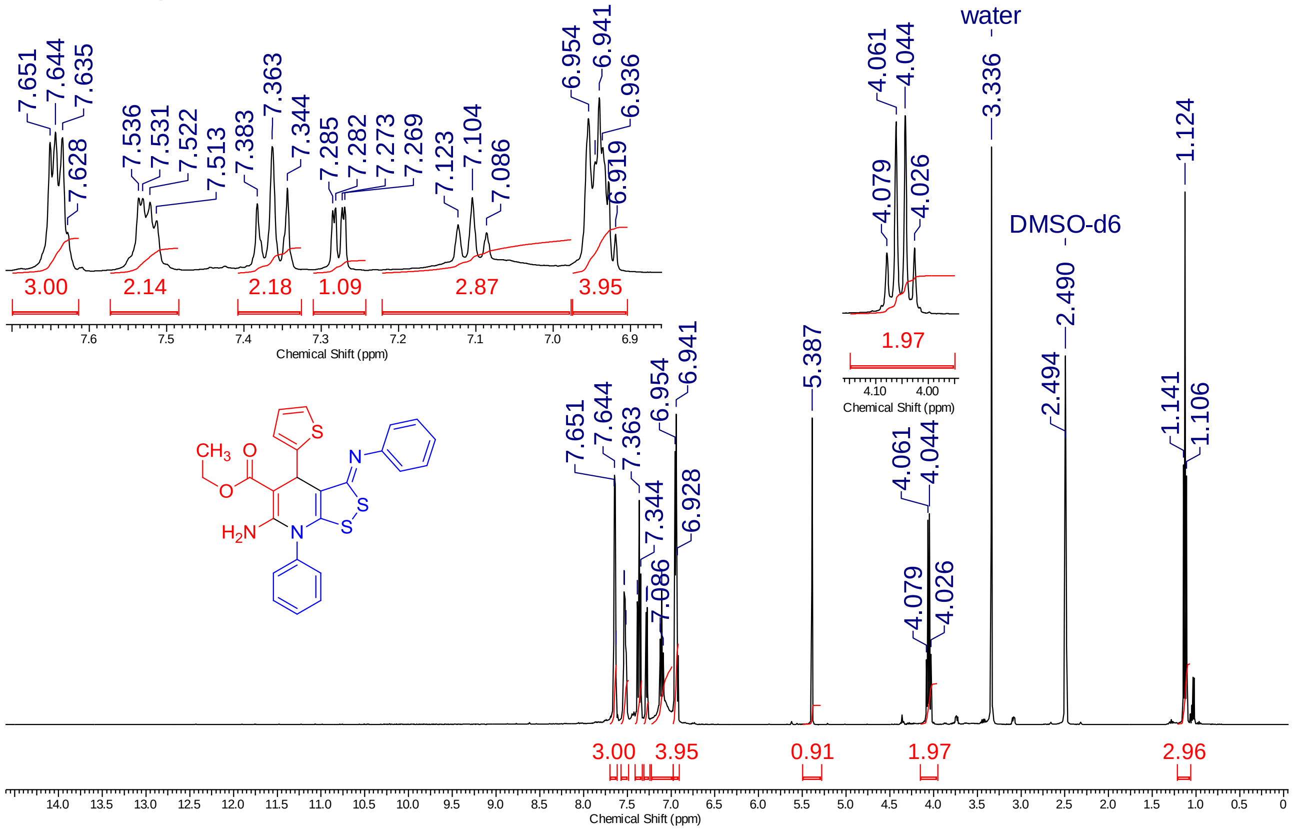

Figure S13. ^{13}C DEPTQ NMR spectrum of dithiolopyridine 15c, DMSO-d$_6$ (101 MHz)

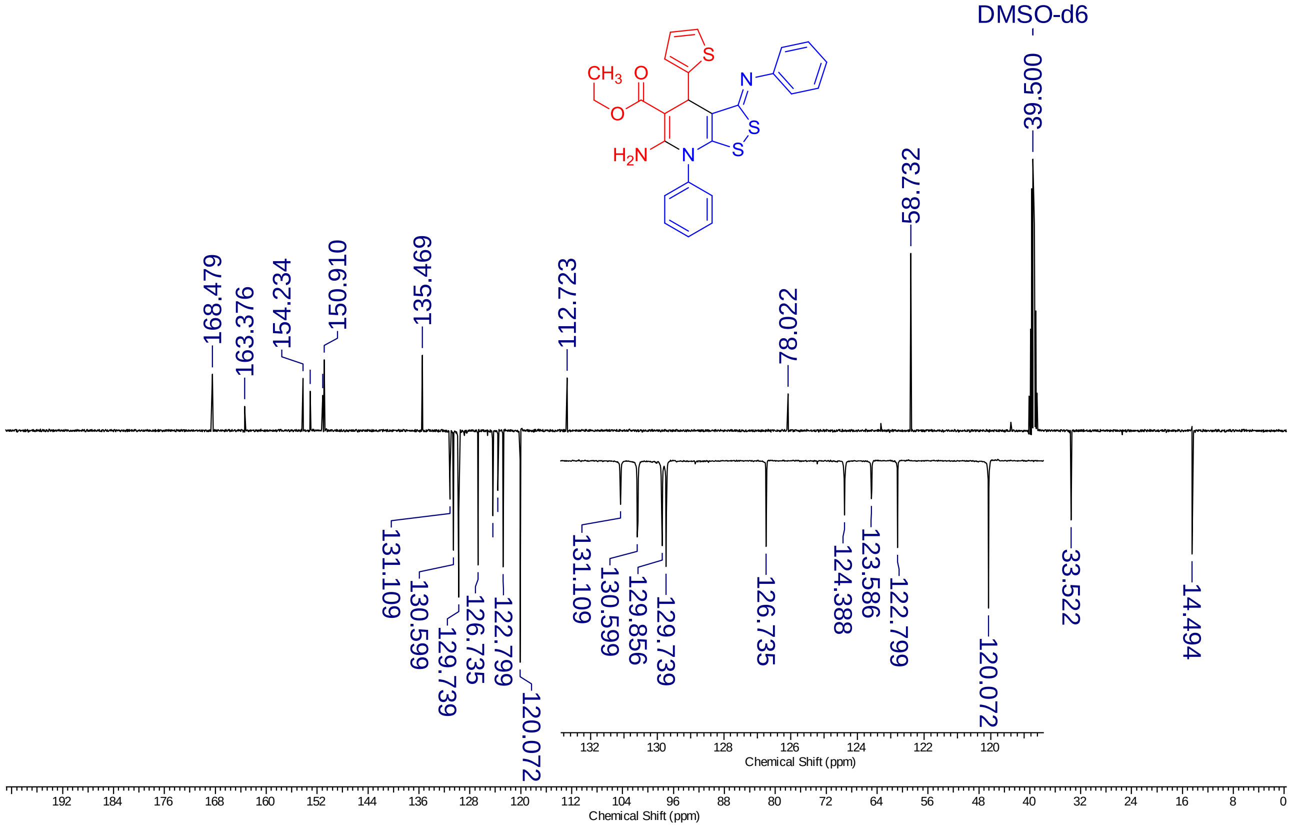

Figure S14. ^{1}H–^{13}C HSQC NMR spectrum of the dithiolopyridine 15c, DMSO-d_6 (400/101 MHz)

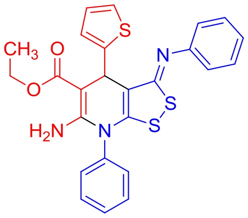

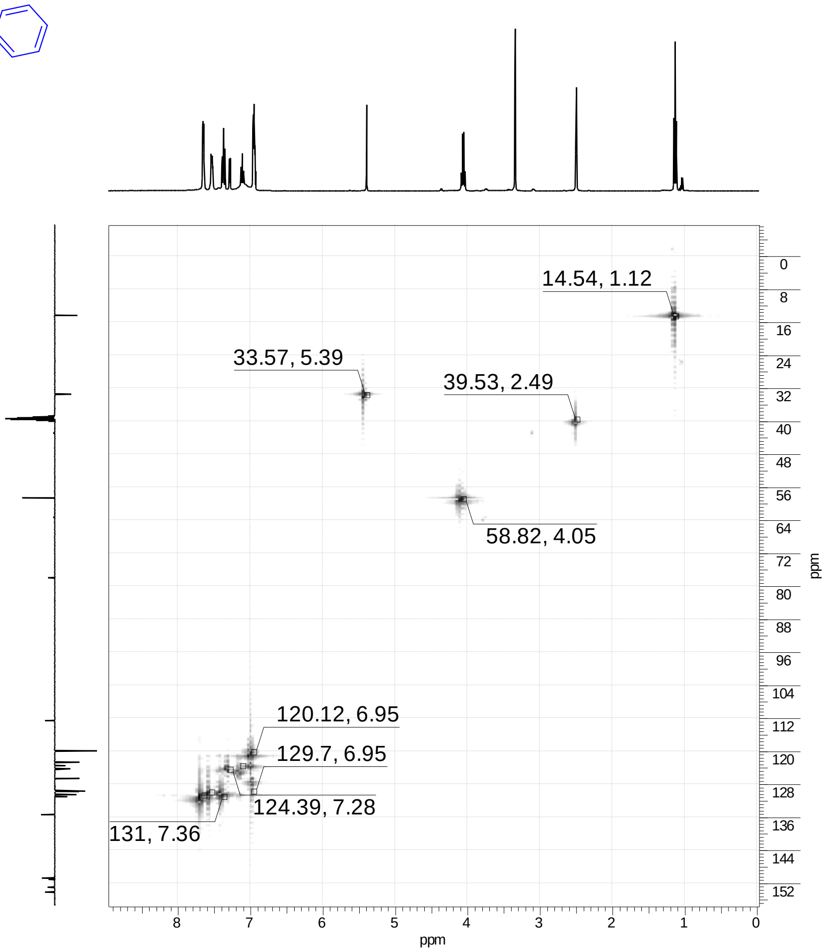

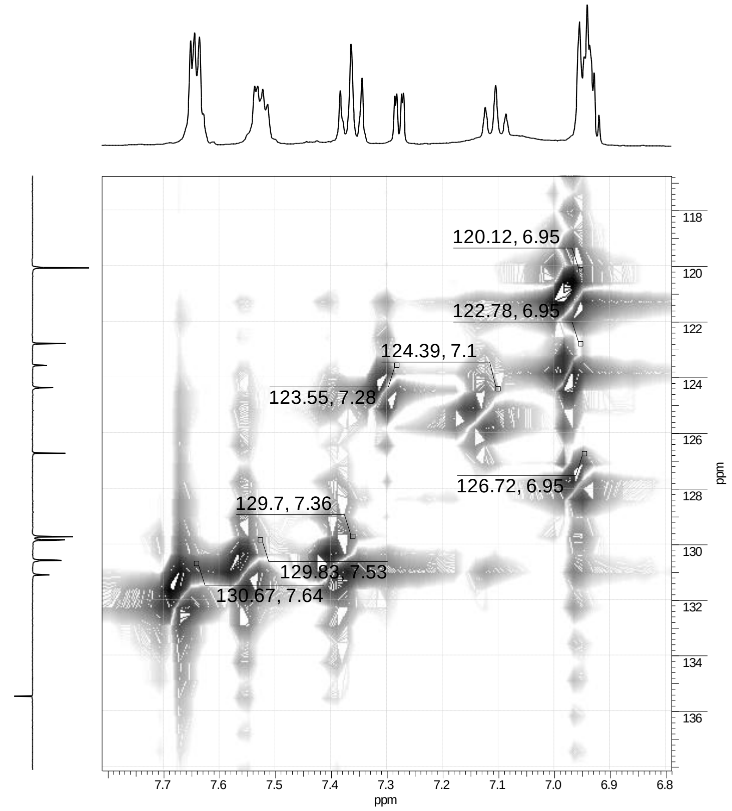

Figure S15. ¹H–¹³C HMBC NMR spectrum of the dithiolopyridine 15c, DMSO-d_6 (400/101 MHz)

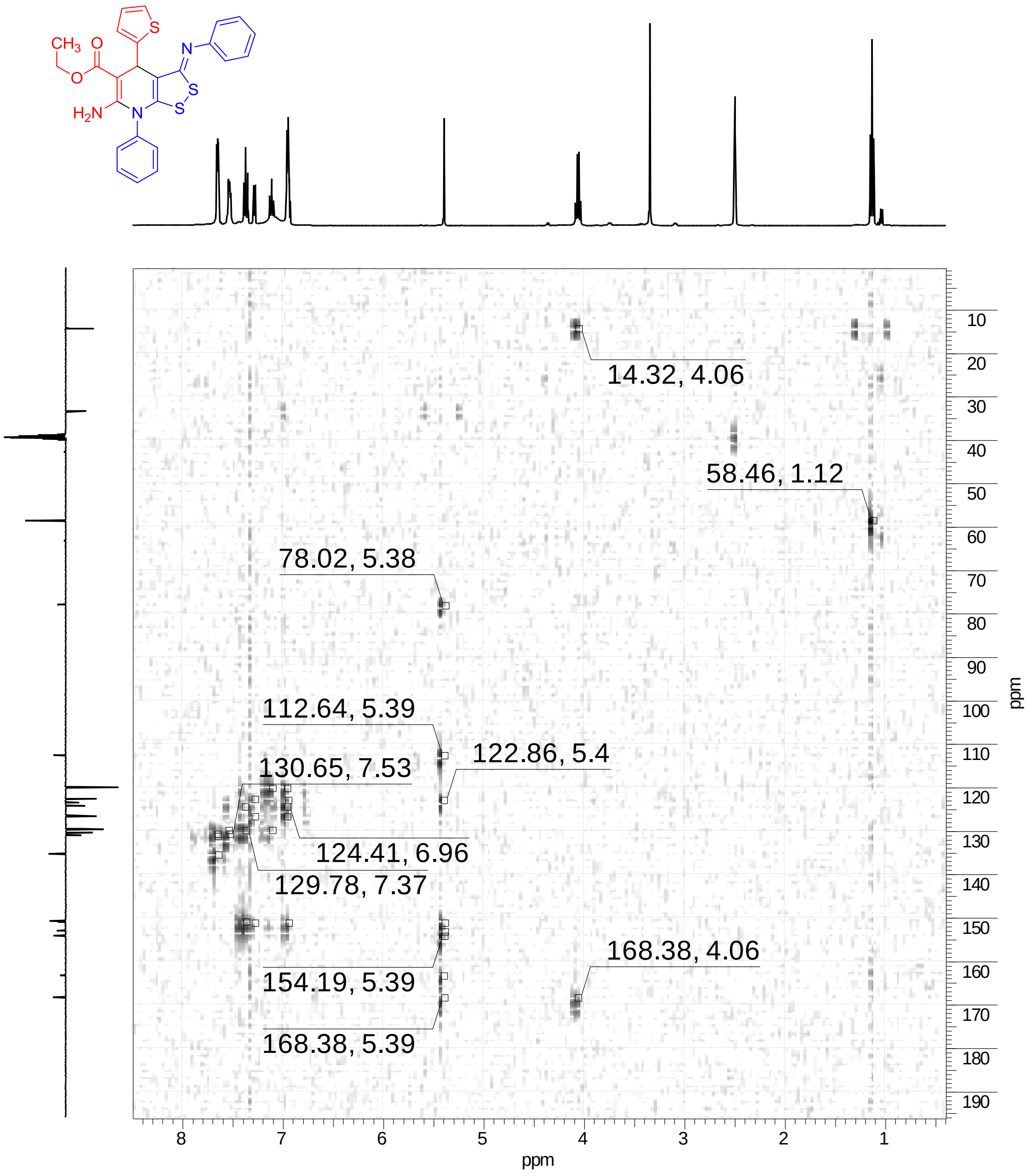

Figure S16. ^{1}H–^{13}C HMBC NMR spectrum of the dithiolopyridine 15c, DMSO-d$_6$ (400/101 MHz) (fragment)

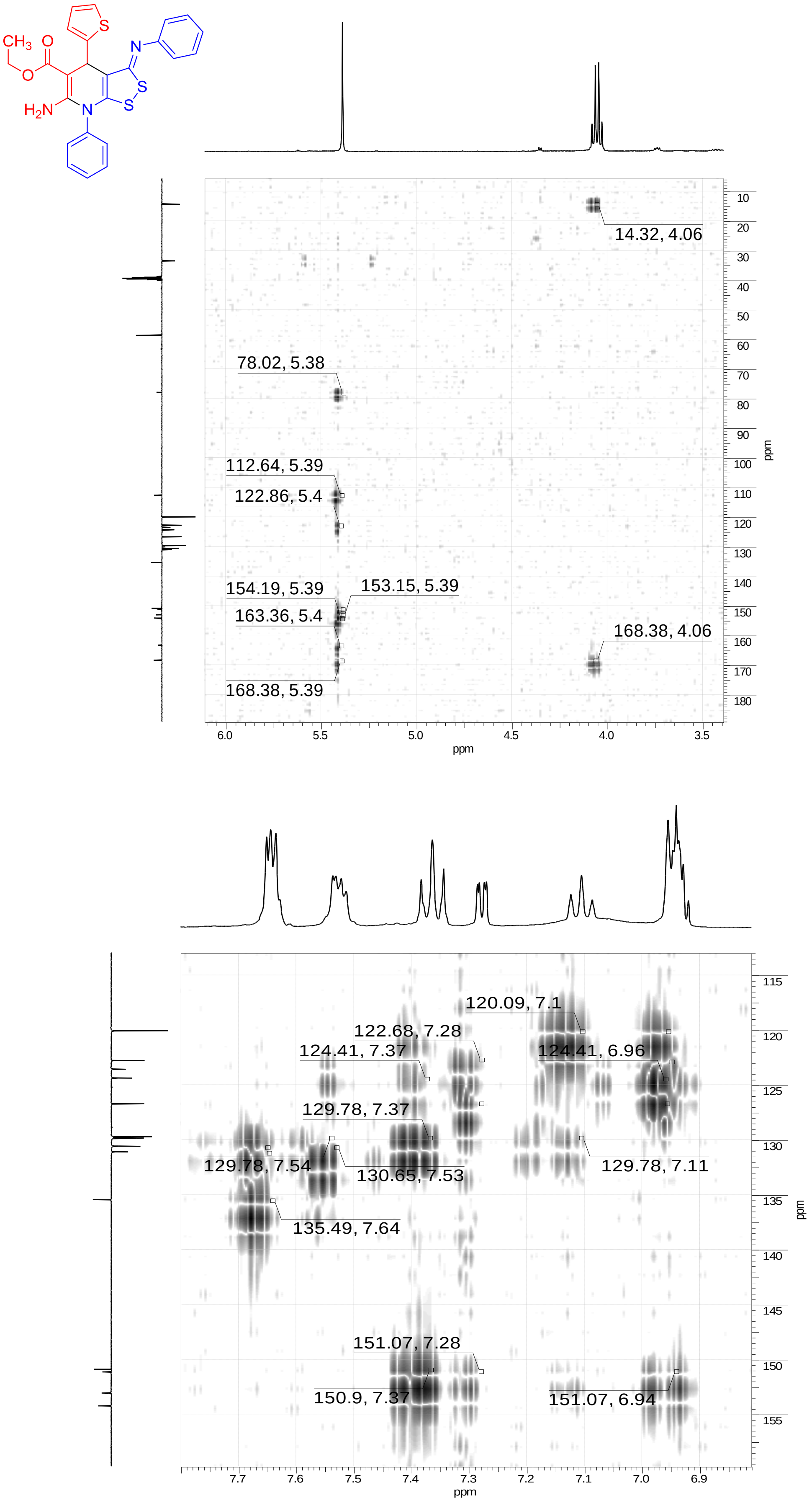

Table S8. The observed correlations in the ^{1}H-^{13}C HSQC and ^{1}H-^{13}C HMBC 2D NMR spectra of dithiolopyridine 15c

^{13}C chemical shifts are given in **red**, ^{1}H shifts – in **blue**

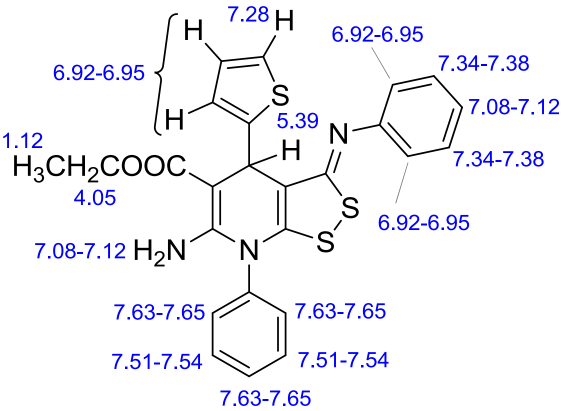

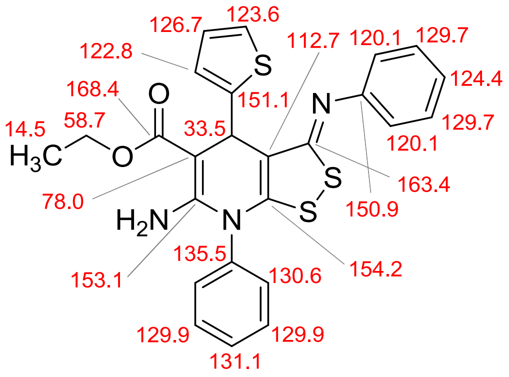

^{1}H NMR shifts, δ, ppm	Correlations in HSQC spectrum, δ, ppm	Correlations in HMBC spectrum, δ, ppm
1.12 (t, ^{3}J = 7.1 Hz, 3H, C<u>H</u>$_3$CH$_2$)	14.5* (CH$_3$)	58.7 (OCH$_2$)
4.05 (q, ^{3}J = 7.1 Hz, 2H, CH$_3$C<u>H</u>$_2$)	58.7 (OCH$_2$)	14.5* (CH$_3$), 168.5 (C=O)
5.39 (s, 1H, H-4)	33.5* (C-4)	78.0 (C-5), 112.7 (C-3a), 122.8* (C-2 thienyl), 151.1 (C-1 thienyl), 153.1 (C-6), 154.2 (C-8a), 163.4 (C=N), 168.5 (C=O)
6.92-6.95 (m, 4H, H-3 H-4 thienyl and H-2 H-6 Ph overlapped)	120.1* (2C, C2 C-6 Ph), 122.8* (C-2 thienyl), 126.7* (C-4 thienyl)	120.1* (2C, C2 C-6 Ph), 122.8* (C-2 thienyl), 124.4* (C-4 Ar), 126.7* (C-4 thienyl), 151.1 (C-1 thienyl)
7.09–7.12 (m, 3H, H-4 Ph and NH$_2$ overlapped)	124.4* (C-4 Ar)	120.1* (2C, C2 C-6 Ph), 129.7* (2C, C-3 C-5 Ph)
7.28 (dd, ^{3}J = 4.9 Hz, ^{4}J = 1.5 Hz, 1H, H-5 2-thienyl)	123.6* (C-5 thienyl)	122.8* (C-2 thienyl), 126.7* (C-4 thienyl), 151.1 (C-1 thienyl)
7.34–7.38 (m, 2H, H-3 H-5 Ph)	129.7* (2C, C-3 C-5 Ph)	124.4* (C-4 Ar), 129.7* (2C, C-3 C-5 Ph), 150.9 (C-1 Ph)
7.51–7.54 (m, 2H, H-3 H-5 Ph)	129.9* (2C, C-3 C-5 Ph)	129.9* (2C, C-3 C-5 Ph), 130.6* (2C, C-2 C-6 Ph)
7.63–7.65 (m, 3H, H-2 H-6 H-4 Ph)	130.6* (2C, C-2 C-6 Ph), 131.1* (C-4 Ph)	130.6* (2C, C-2 C-6 Ph), 131.1* (C-4 Ph), 135.5 (C-1 Ph)

*Signals with a negative phase.

Figure S17. FTIR spectrum of dithiolopyridine 15d

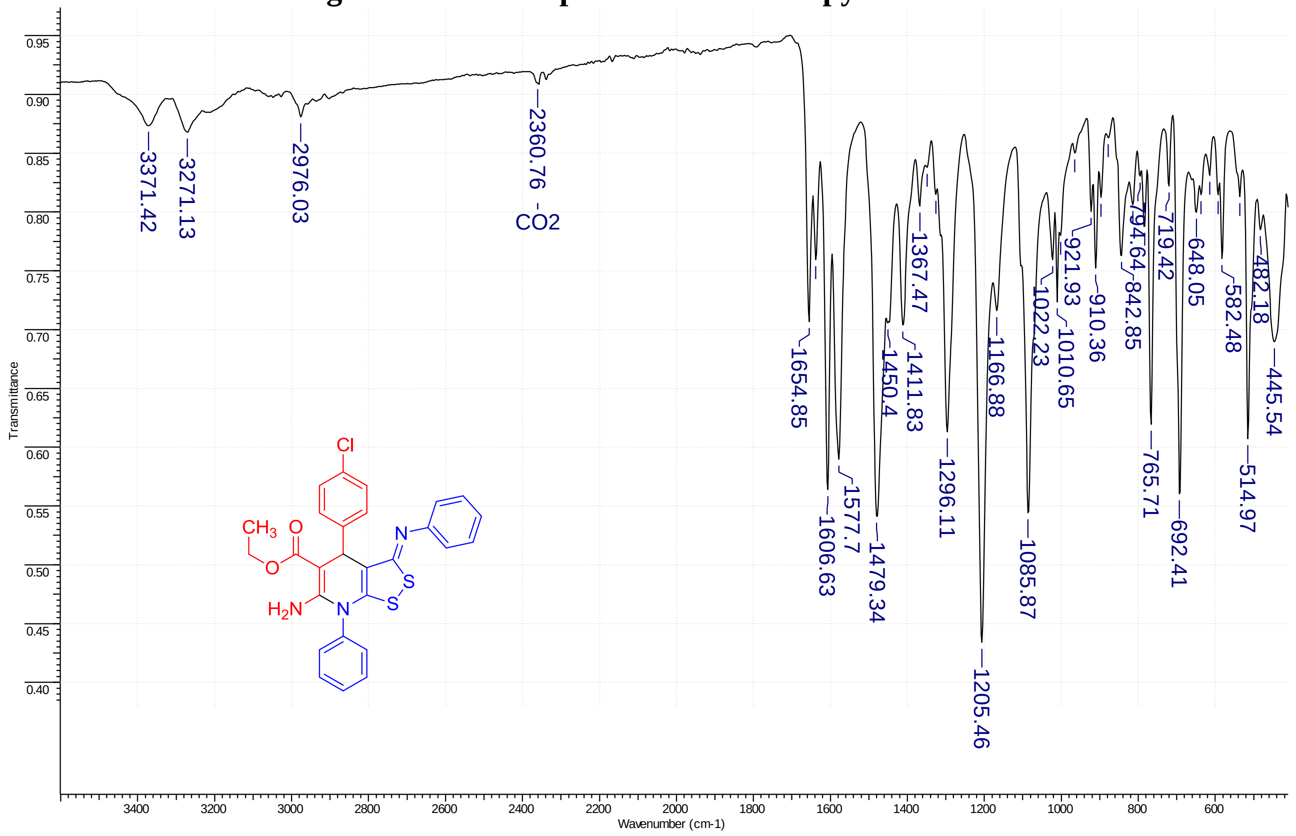

Figure S18. ^{1}H NMR spectrum of dithiolopyridine 15d, DMSO-d_6 (400 MHz)

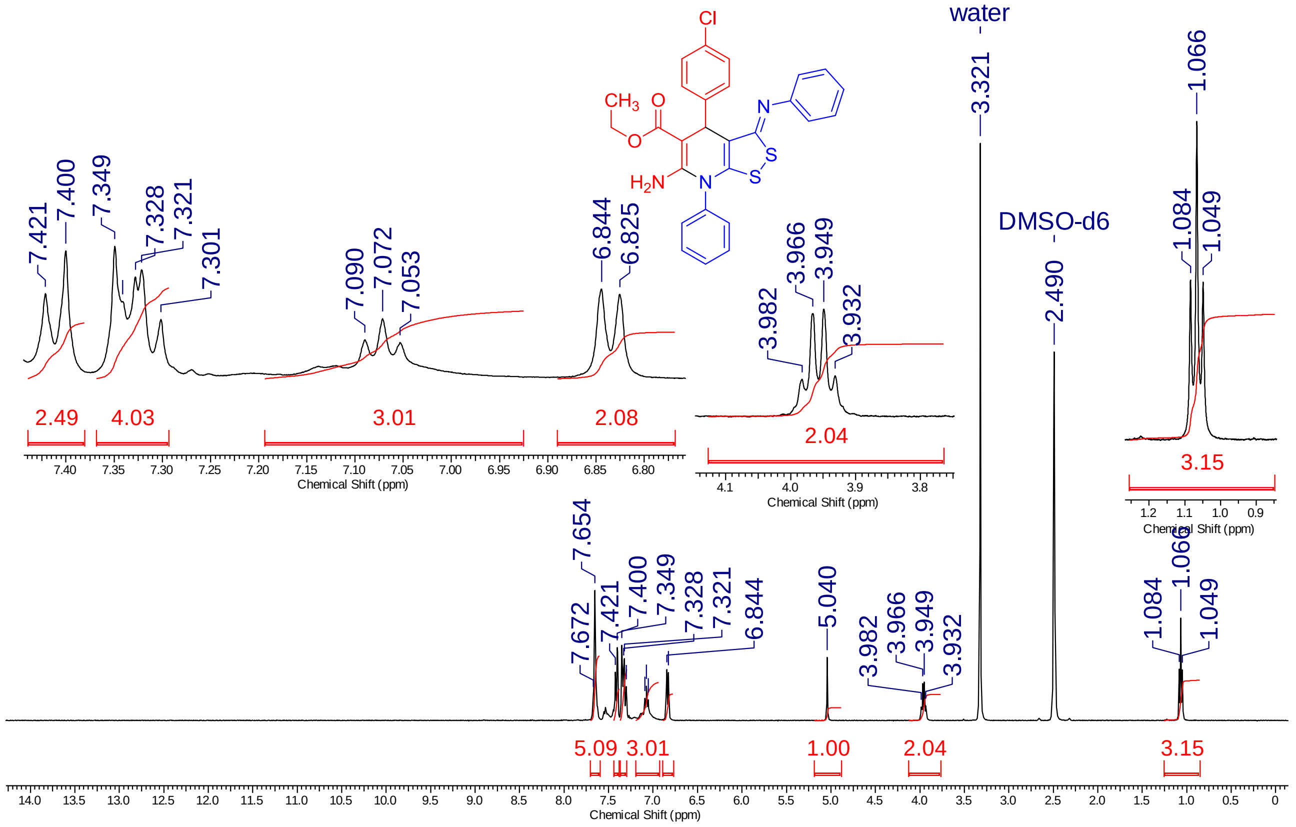

Figure S19. ^{13}**C DEPTQ NMR spectrum of dithiolopyridine 15d, DMSO-d$_6$ (101 MHz)**

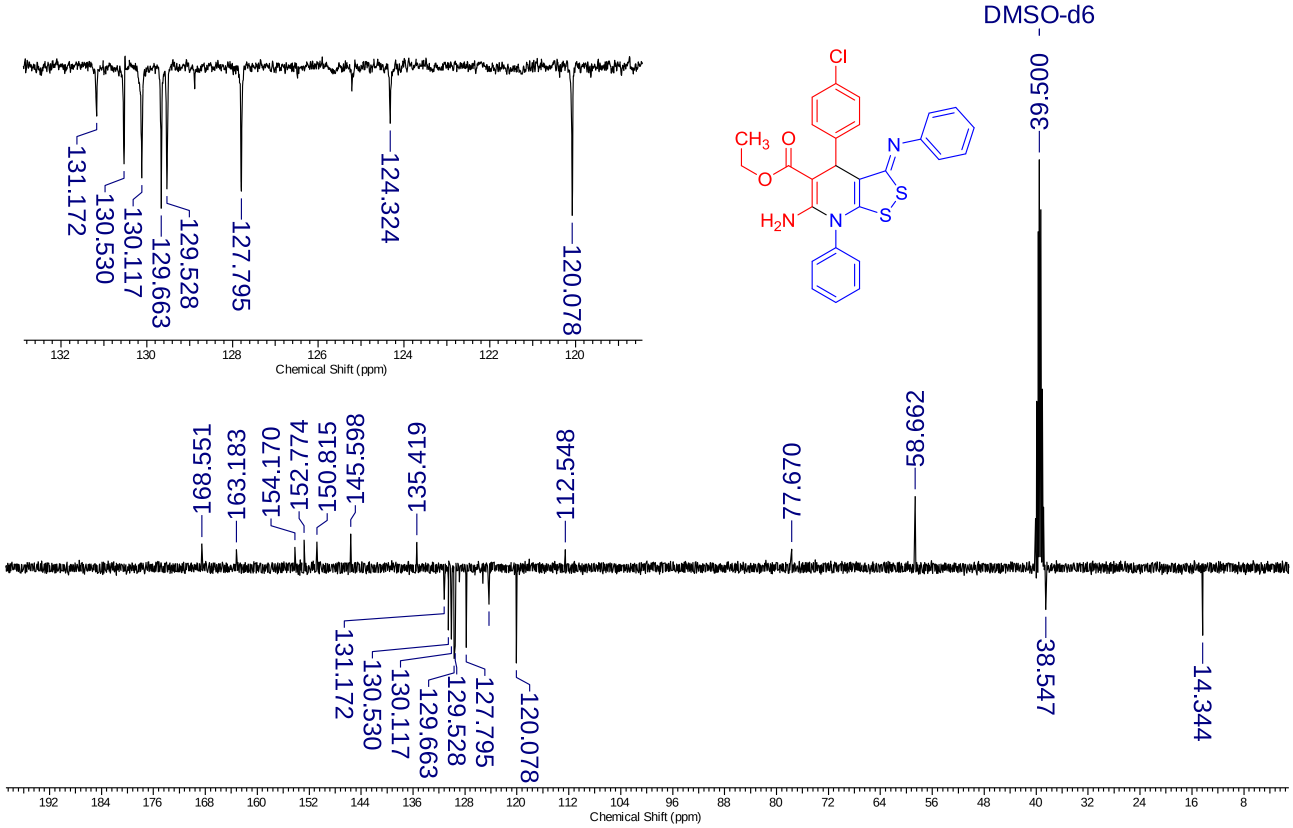

Figure S20. FTIR spectrum of dithiolopyridine 15f

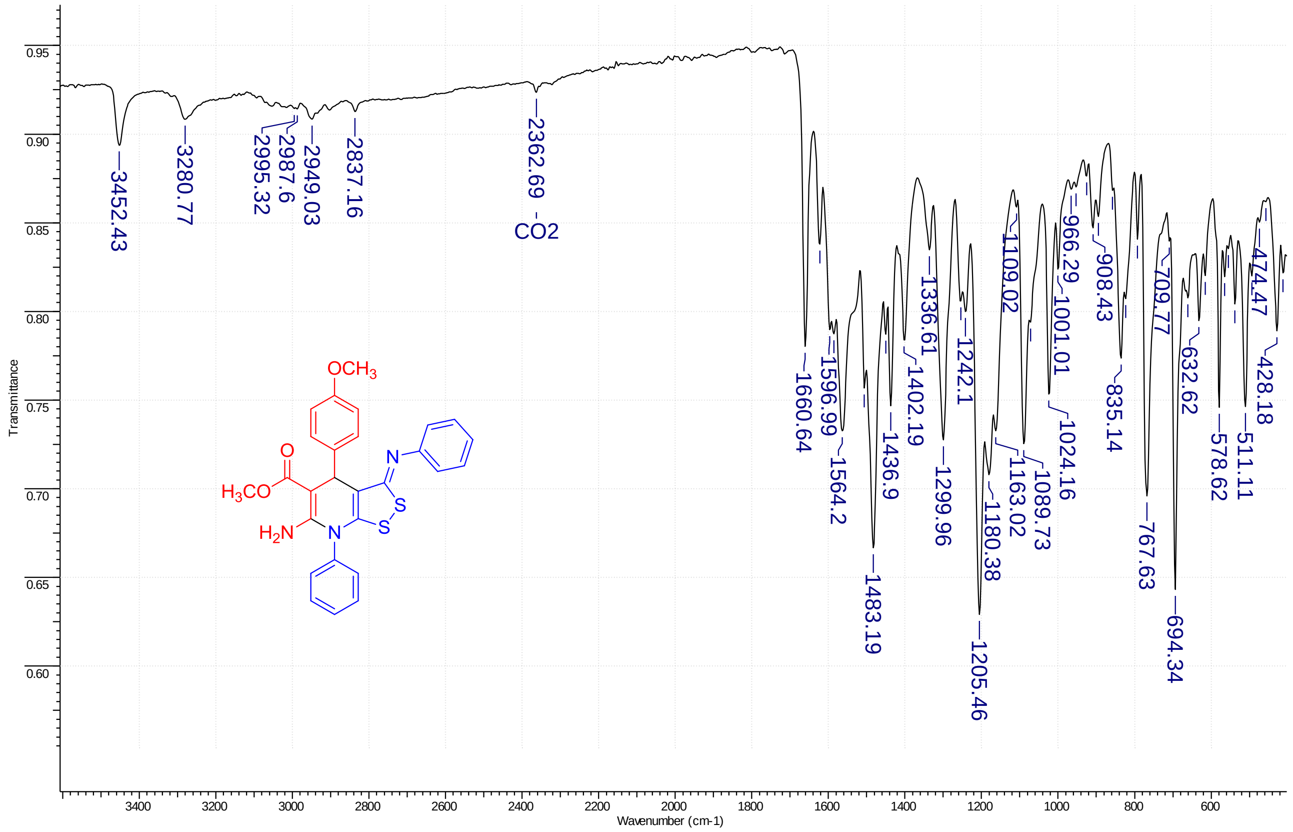

Figure S21. ^{1}**H NMR spectrum of dithiolopyridine 15f, DMSO-d$_6$ (400 MHz)**

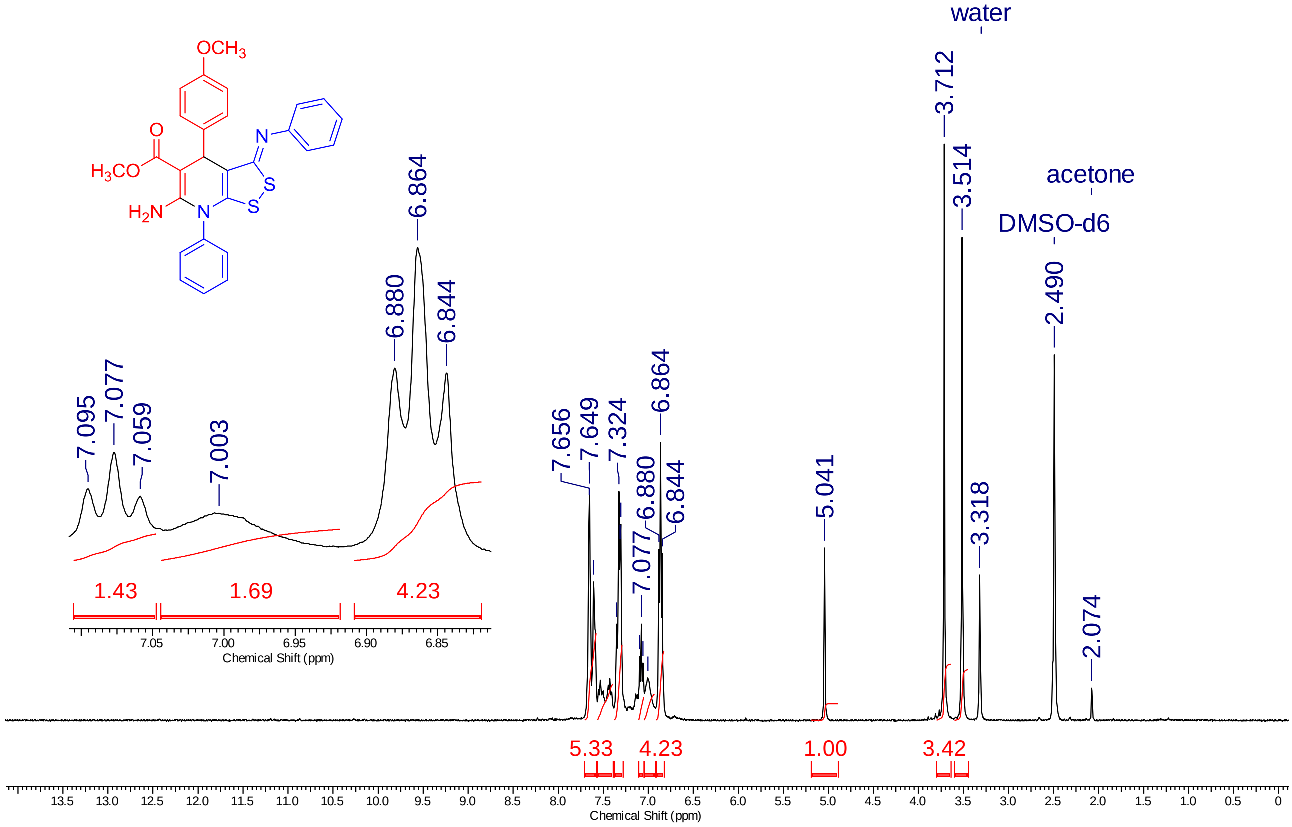

Figure S22. ^{13}**C DEPTQ NMR spectrum of dithiolopyridine 15f, DMSO-d$_6$ (101 MHz)**

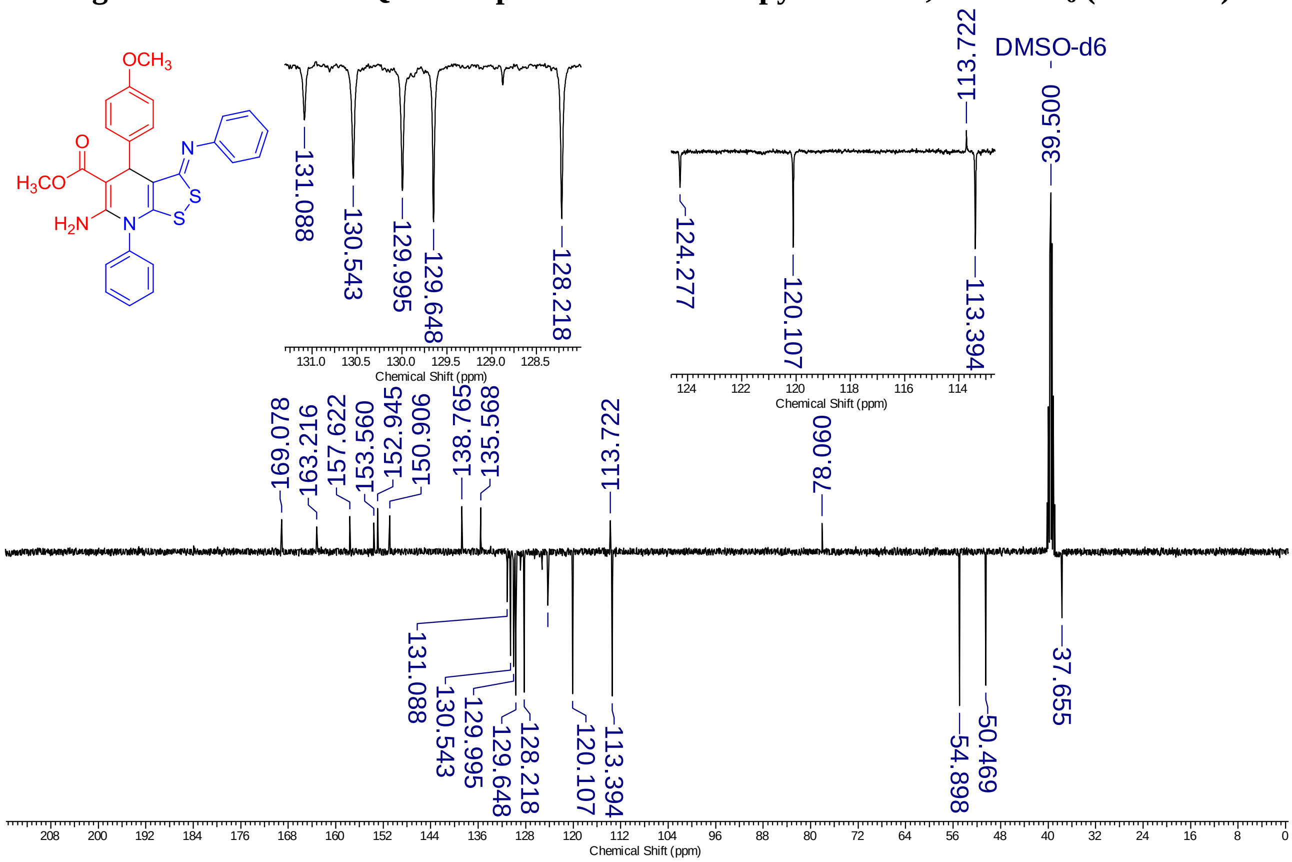

Figure S23. ^{1}H–^{13}C HSQC NMR spectrum of the dithiolopyridine 15f, DMSO-d_6 (400/101 MHz)

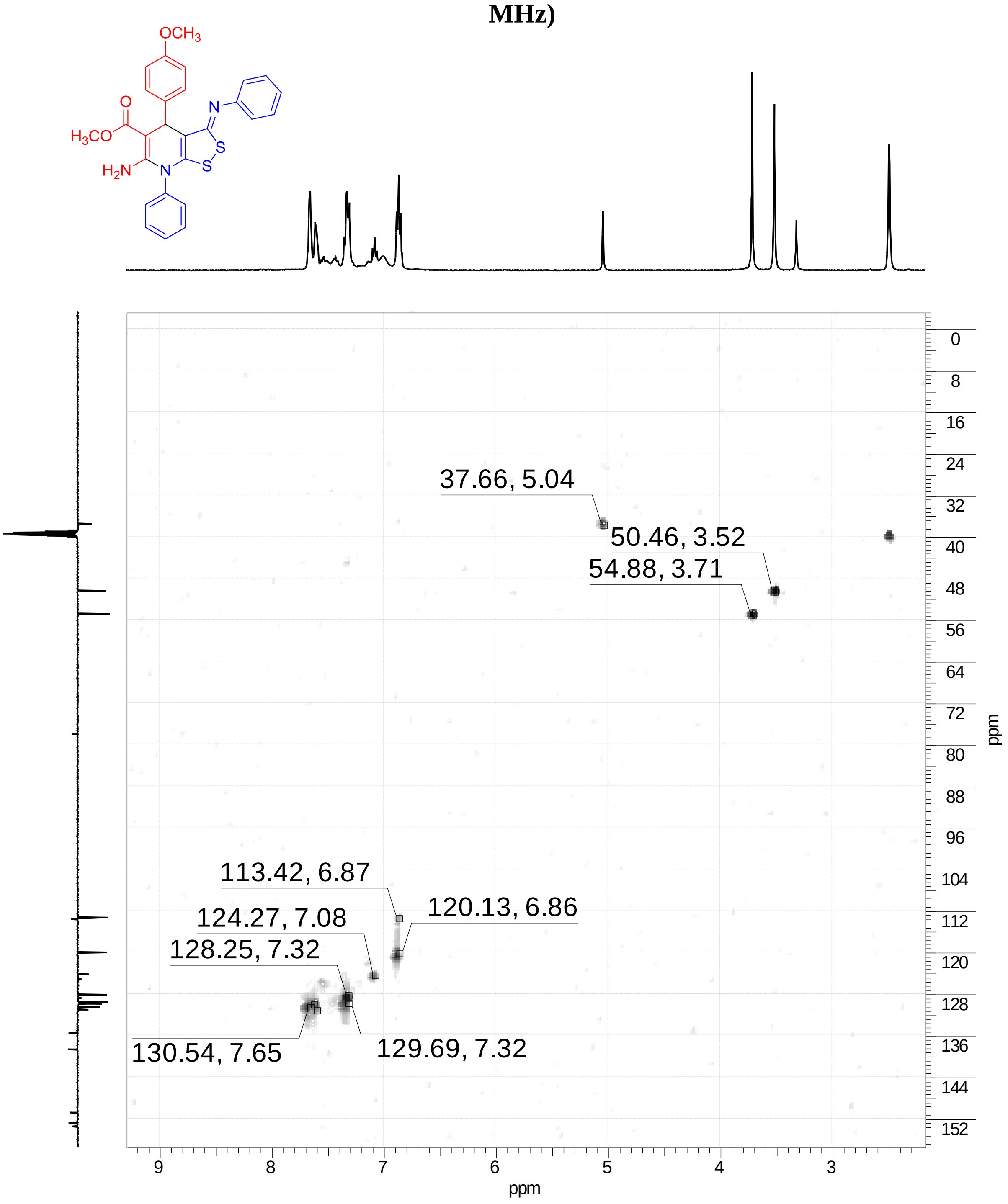

Figure S24. ^{1}H–^{13}C **HMBC NMR spectrum of the dithiolopyridine 15f, DMSO-d_6 (400/101 MHz)**

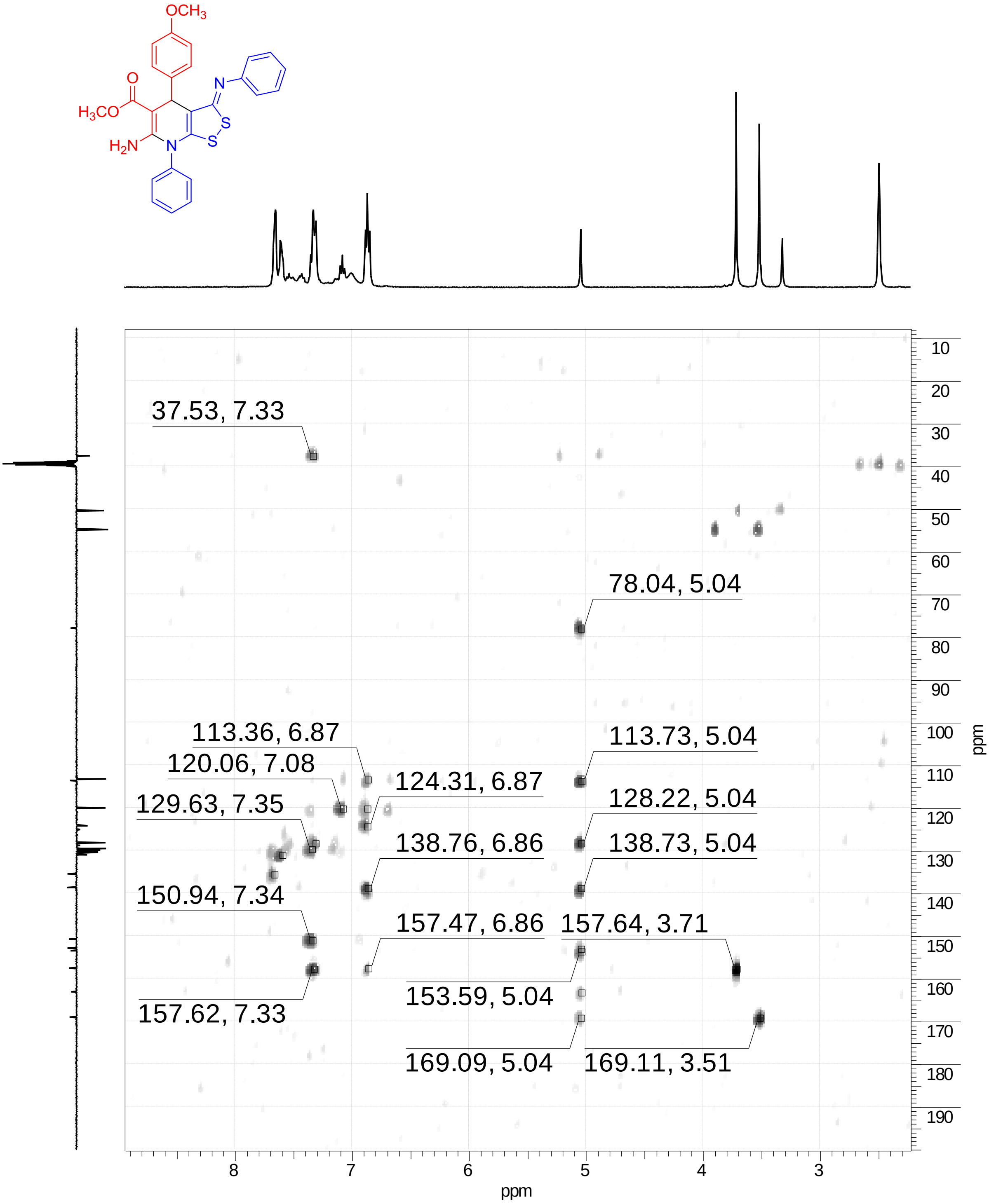

Figure S25. ^1H–^{13}C **HMBC NMR spectrum of the dithiolopyridine 15f, DMSO-d$_6$ (400/101 MHz) (*fragment*)**

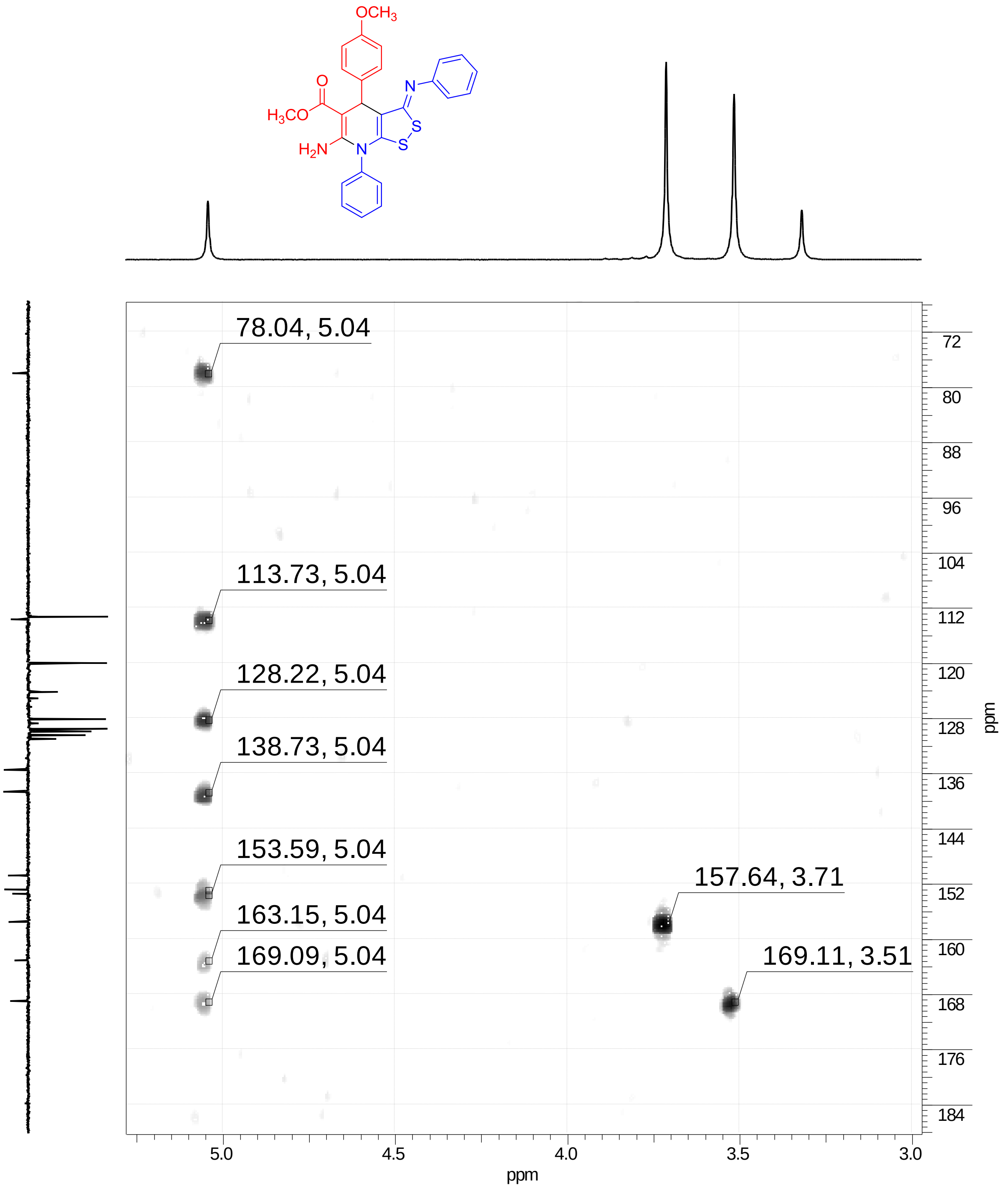

Table S9. The observed correlations in the ^{1}H-^{13}C HSQC and ^{1}H-^{13}C HMBC 2D NMR spectra of dithiolopyridine 15f

^{13}C chemical shifts are given in **red**, ^{1}H shifts – in **blue**

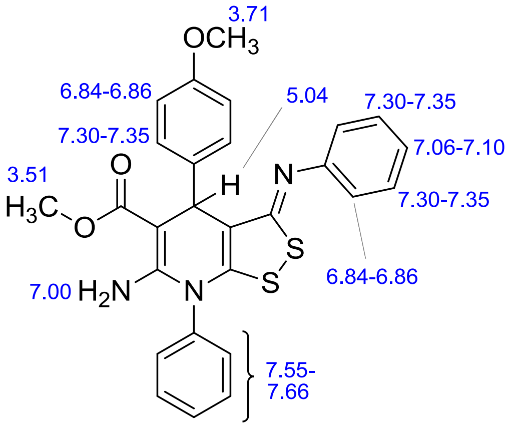

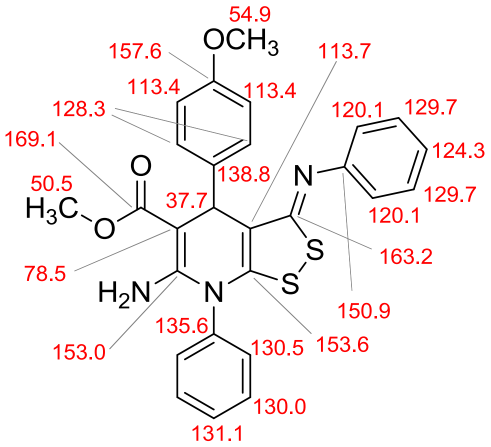

^{1}H NMR shifts, δ, ppm	Correlations in HSQC spectrum, δ, ppm	Correlations in HMBC spectrum, δ, ppm
3.51 (s, 3H, COOMe)	50.5* ($CO_2\underline{C}H_3$)	169.1 (C=O)
3.71 (s, 3H, MeO–Ar)	54.9* (OCH_3)	157.6 (C–OMe)
5.04 (s, 1H, H-4)	37.7* (C-4)	78.1 (C-5), 113.7 (C-3a), 128.3* (2C, CH Ar), 138.8 (C-1 Ar), 153.0 (C-6), 153.6 (C-8a), 163.2 (C=N), 169.1 (C=O)
6.84–6.88 (m, 4H, H Ar)	113.4* (2C, CH Ar) 120.1* (2C, CH Ar)	113.4* (2C, CH Ar), 120.1* (2C, CH Ar), 124.3* (CH Ar), 138.8 (C-1 Ar), 157.6 (C–OMe)
7.00 (very br s, 2H, NH_2)	–	–
7.06–7.10 (m, 1H, H-4 Ph)	124.3* (CH Ar)	120.1* (2C, CH Ar)
7.30–7.35 (m, 4H, H Ar)	128.3* (2C, CH Ar), 129.7* (2C, CH Ar)	37.7* (C-4), 128.3* (2C, CH Ar), 129.7* (2C, CH Ar), 150.9 (C-1 Ph), 157.6 (C–OMe)
7.55–7.66 (m, 5H, H Ar)	130.0* (CH Ar), 130.5* (CH Ar), 131.1* (CH Ar)	130.0* (CH Ar), 130.5* (CH Ar), 131.1* (CH Ar), 135.6 (C-1 Ph)

*Signals with a negative phase.

Figure S26. HRMS spectrum of dithiolopyridine 15a

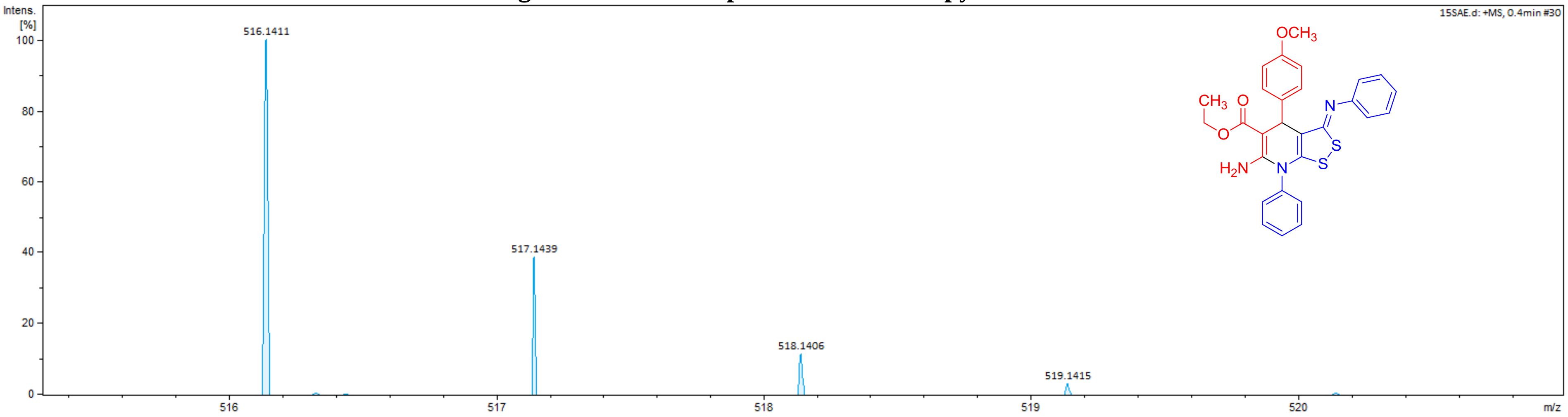

Figure S27. HRMS spectrum of dithiolopyridine 15b

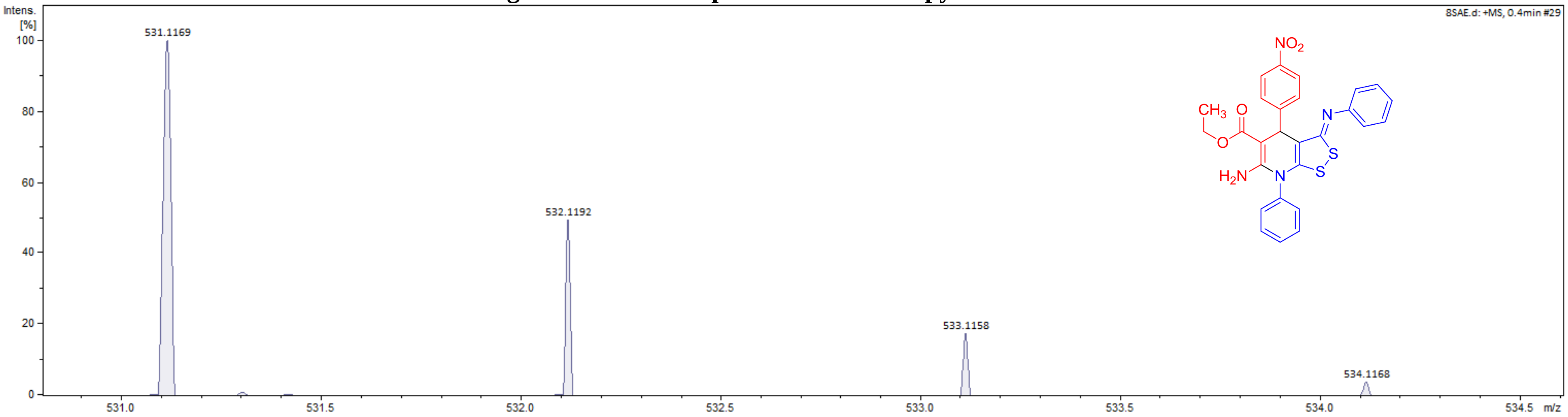

Figure S28. HRMS spectrum of dithiolopyridine 15c

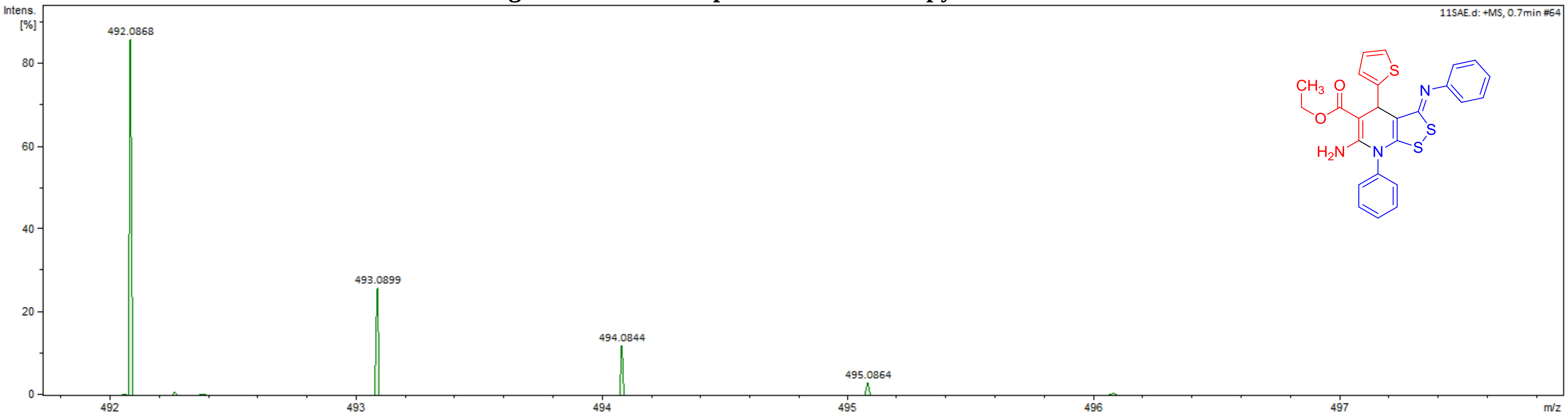

Figure S29. HRMS spectrum of dithiolopyridine 15d

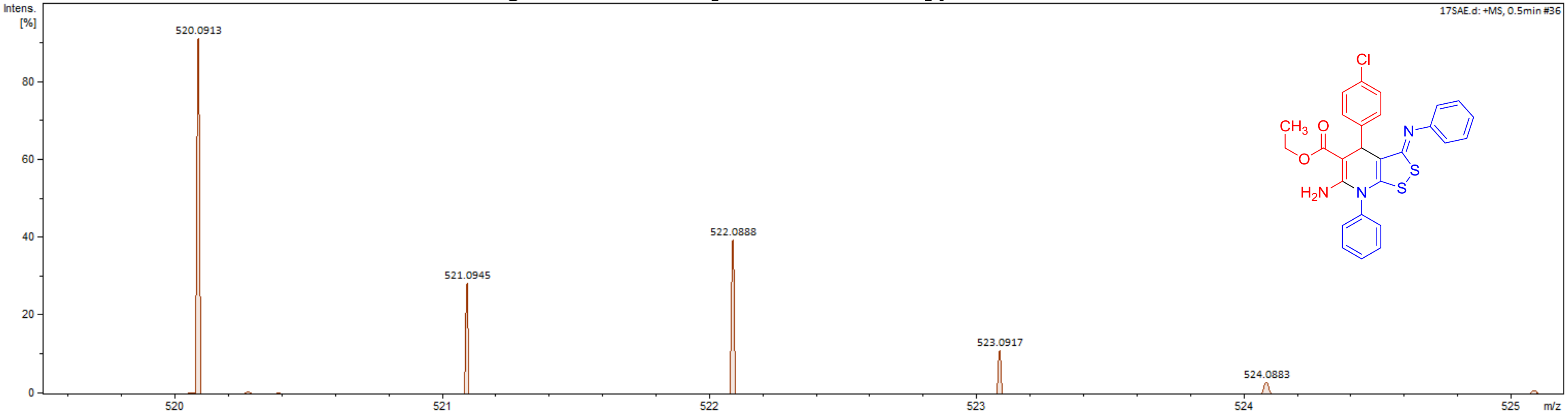

Figure S30. HRMS spectrum of dithiolopyridine 15e

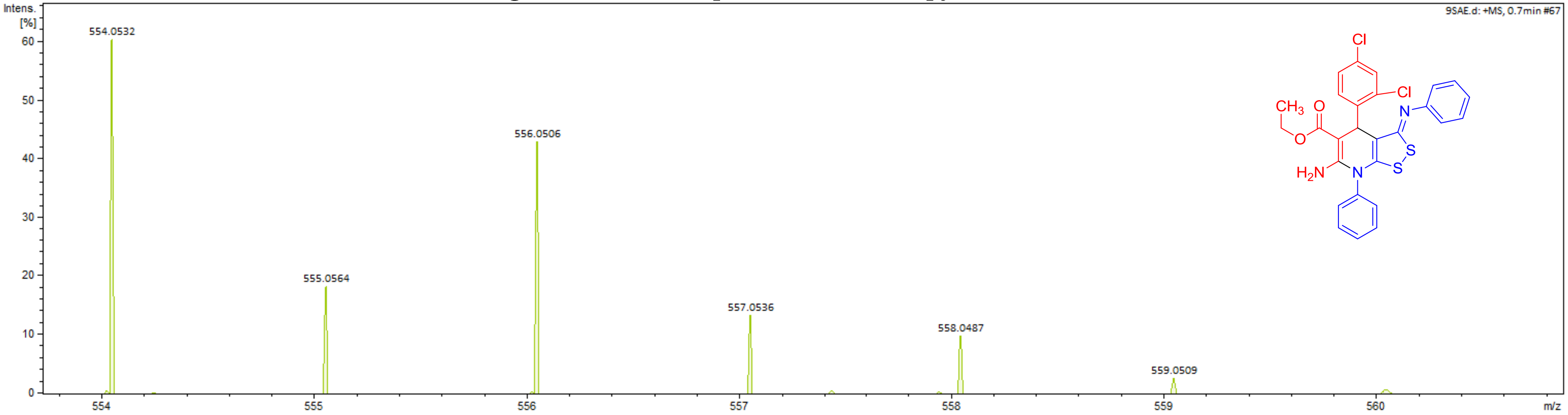

Figure S31. HRMS spectrum of dithiolopyridine 15f

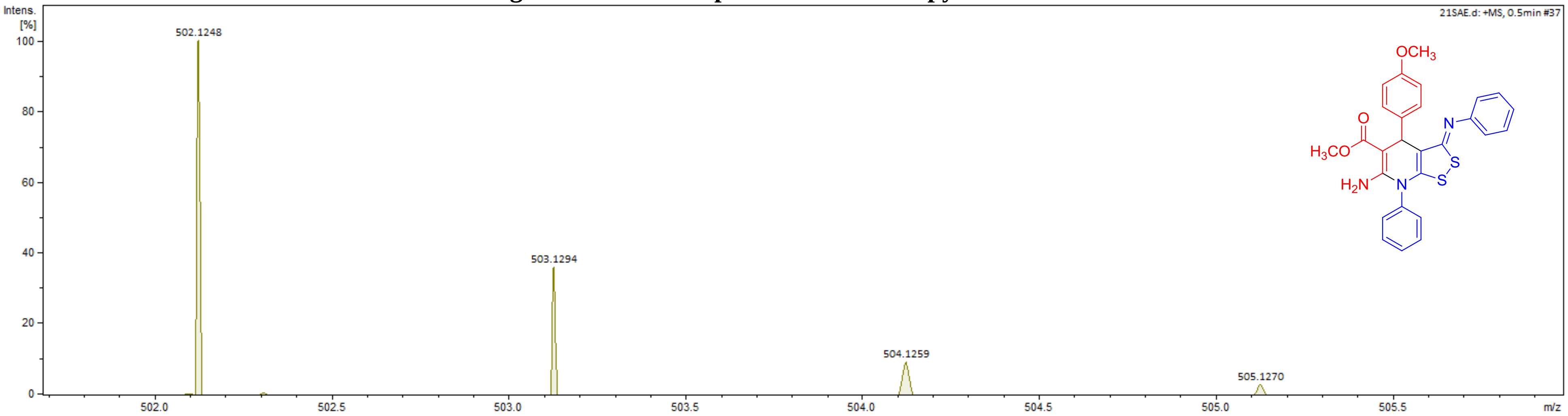

Supplement: Supplementary file 1 [file molecules-28-00609-s001.zip › molecules-2123635-supplementary.pdf]
